# Molecular constituents of the extracellular matrix in rat liver mounting a hepatic progenitor cell response for tissue repair

**DOI:** 10.1186/1755-1536-6-21

**Published:** 2013-12-20

**Authors:** Peter Siig Vestentoft, Peter Jelnes, Jesper B Andersen, Thi Anh Thu Tran, Tenna Jørgensen, Morten Rasmussen, Jette Bornholdt, Lene Melsæther Grøvdal, Charlotte Harken Jensen, Lotte Katrine Vogel, Snorri S Thorgeirsson, Hanne Cathrine Bisgaard

**Affiliations:** 1 Department of Cellular and Molecular Medicine, Faculty of Health and Medical Sciences, University of Copenhagen, Blegdamsvej 3, DK-2200 Copenhagen, Denmark; 2 Laboratory of Experimental Carcinogenesis, National Cancer Institute, National Institutes of Health, Bethesda, MD, USA; 3 The Novo Nordisk Foundation Center for Basic Metabolic Research, Faculty of Health Sciences, University of Copenhagen, Copenhagen, Denmark; 4 Laboratory of Molecular and Cellular Cardiology, Department of Clinical Biochemistry and Pharmacology, Odense University Hospital, Odense, Denmark; 5 Department of Cardiovascular and Renal Research, University of Southern Denmark, Odense, Denmark

**Keywords:** Ductular reaction, Extracellular matrix constituents, First and second tiers of defense, Hepatic progenitor (oval) cell reaction, Liver injury and repair, Three-dimensional reconstruction

## Abstract

**Background:**

Tissue repair in the adult mammalian liver occurs in two distinct processes, referred to as the first and second tiers of defense. We undertook to characterize the changes in molecular constituents of the extracellular matrix when hepatic progenitor cells (HPCs) respond in a second tier of defense to liver injury.

**Results:**

We used transcriptional profiling on rat livers responding by a first tier (surgical removal of 70% of the liver mass (PHx protocol)) and a second tier (70% hepatectomy combined with exposure to 2-acetylaminofluorene (AAF/PHx protocol)) of defense to liver injury and compared the transcriptional signatures in untreated rat liver (control) with those from livers of day 1, day 5 and day 9 post hepatectomy in both protocols. Numerous transcripts encoding specific subunits of collagens, laminins, integrins, and various other extracellular matrix structural components were differentially up- or down-modulated (*P* < 0.01). The levels of a number of transcripts were significantly up-modulated, mainly in the second tier of defense (*Agrn, Bgn, Fbn1, Col4a1, Col8a1, Col9a3, Lama5, Lamb1, Lamb2, Itga4, Igtb2, Itgb4, Itgb6, Nid2*), and their signal intensities showed a strong or very strong correlation with *Krt1-*19, a well-established marker of a ductular/HPC reaction. Furthermore, a significant up-modulation and very strong correlation between the transcriptional profiles of *Krt1-19* and *St14* encoding matriptase, a component of a novel protease system, was found in the second tier of defense. Real-time PCR confirmed the modulation of *St14* transcript levels and strong correlation to *Krt-19* and also showed a significant up-modulation and strong correlation to *Spint1* encoding HAI-1, a cognate inhibitor of matriptase. Immunodetection and three-dimensional reconstructions showed that laminin, Collagen1a1, agrin and nidogen1 surrounded bile ducts, proliferating cholangiocytes, and HPCs in ductular reactions regardless of the nature of defense. Similarly, matriptase and HAI-1 were expressed in cholangiocytes regardless of the tier of defense, but in the second tier of defense, a subpopulation of HPCs in ductular reactions co-expressed HAI-1 and the fetal hepatocyte marker Dlk1.

**Conclusion:**

Transcriptional profiling and immunodetection, including three-dimensional reconstruction, generated a detailed overview of the extracellular matrix constituents expressed in a second tier of defense to liver injury.

## Background

Morphologically, the adult liver is characterized by two epithelial tissue structures, hepatic cords, and bile ducts. During development, hepatocytes and cholangiocytes, the cellular epithelial component of the biliary tree, differentiate from hepatoblasts and the latter form portal tubular structures in a complex remodeling process. The adult liver possesses an exceptional regenerative capacity in response to injury, which can be accomplished through two distinct processes, referred to as the first and second tiers of defense. In the first tier of defense, the liver can regrow to its original mass and obtain full functional capacity through division of normally quiescent hepatocytes and cholangiocytes [1]. However, when hepatocytic division is compromised, proliferation of epithelial cells in the canal of Hering, the most distal part of the biliary tree, is observed. Because of their ability to proliferate extensively, express proteins such as α-fetoprotein (Afp) and Delta-like 1 homolog (Dlk1), which are normally only found in hepatoblasts and hepatocytes during liver development, and to differentiate into fully functional hepatocytes or cholangiocytes, these cells are regarded as proliferating hepatic progenitor cells (HPCs) and constitute the second tier of defense in the reaction to injury. The canal of Hering is, therefore, thought to comprise the adult hepatic progenitor cell niche, a protective microenvironment that serves to maintain and regulate HPC activity [2-6].

As with arrangements defined in stem cell niches of other organs [7,8], the hepatic progenitor cell niche is thought to be structurally composed of a facultative stem or progenitor cell population situated on a basal lamina consisting of a highly crosslinked extracellular matrix (ECM) of collagens, laminins, and nidogens. Together with other structural constituents, proteases and their inhibitors, as well as associated molecules, an ECM microenvironment is created, in which intimate contact of HPCs with supporting cells, such as stellate cells, myofibroblasts and macrophages, occurs [2,3,6]. Upon massive injury to hepatocytes, the niche is thought to respond by changing the molecular composition of active signaling pathways and remodeling the ECM microenvironment affecting both HPCs and supporting cells. A number of molecules involved in modulating this response have been identified [9]. Interestingly, they include several important players in ECM remodeling in liver fibrosis, such as connective tissue growth factor [10,11], transforming growth factor-β [12], which is active in ECM deposition, and matrix metalloproteinases 2, 9, and 12 (MMP-2, MMP-9 and MMP-12) and their inhibitor, tissue inhibitor of metalloproteinase type 1 (TIMP-1), which is involved in altered matrix degradation [13,14]. Furthermore, activation of ECM-producing cells and ECM deposition are reported to occur as an initial phase prior to HPC expansion in the injured liver, and in front of HPCs along the porto-veinous gradient of lobular invasion [15]. Subsequently, a key role for laminin in controlling the fate of HPCs was demonstrated with isolated primary rodent HPCs seeded on different matrixes, including collagen I, collagen IV, laminin, and fibronectin, where only interaction with laminin was able to promote or sustain expression of biliary or HPC genes [6]. Altogether, these studies support the hypothesis of a fundamental role for the ECM in establishing specific hepatic microenvironments or niches to regulate the response of HPCs to tissue injury. However, the exact molecular composition of the ECM involved in a HPC response has yet to be reckoned.

This study was undertaken to characterize the molecular constituents of the remodeling extracellular matrix when the hepatic progenitor cell niche responds to liver injury. For this purpose, we applied the following strategy. First, we compiled a hypothetical list of constituents involved in extracellular matrix remodeling based on existing literature (see Table 1). The constituents were based on four groups of molecules: (i) basal lamina and ECM structural constituents, including agrin, biglycan, decorin, elastin, fibrillin, fibronectin, fibulin, collagens, laminins, integrins, nidogens, and SPARC (secreted protein, acidic, cysteine-rich); (ii) ECM proteases and inhibitors, including metalloproteinases (MMPs) and their inhibitors (TIMPs); (iii) other ECM associated molecules, including glypicans and syndecans; and (iv) a novel protease system, composed of, among others, matriptase and its cognate inhibitors, hepatocyte growth factor activator inhibitor (HAI)-1and HAI -2.

**Table 1:** Identification of ECM constituents modulated in first and second tiers of defense upon hepatic injury

**Protein name**	**Gene symbol**	**Modulation of transcriptional profile**		**Product–moment correlation, coefficient *r***
		**First tier of defense using the PHx protocol as model system**	**Second tier of defense using the AAF/PHx protocol as model system**	**Krt1-19-geneX in second tier of defense using the AAF/PHx protocol as model system**
**Hepatic progenitor cell markers**				
Keratin 19	*Krt1-19*	⇅	↑ *	+++++ M
α-Fetoprotein	*Afp*	⇅	↑ *	+++++ M
Epithelial cell adhesion molecule, EpCam	*Tacstd1*	⇅	↑ *	+++++ M
Delta-like homolog 1	*Dlk1*	⇅	↑ *	+++++ M
Deleted in malignant brain tumor 1	*Dmbt1*	⇅	↑ *	+++++ M
Glutathione S-transferase, pi	*Gstp1*	⇅	↑ *	+++++ M
Gap junction protein, α1 (Cx43)	*Gja1*	⇅	↑ *	+++++ M
Prominin, CD133	*Prom1*	⇅	↑ *	+++++ M
CD24	*Cd 24*	⇅	↑ *	+++++ M
Vimentin	*Vim*	⇅	↑ *	+++++ M
Proliferating cell nuclear antigen	*PCNA*	↑ *	↑	+++++
**Mesenchymal cell markers**				
Desmin	*Des*	↑	↑	+++
S100 calcium-binding protein A4	*S100a4*	↑	↑	++++
Secreted phosphoprotein 1 (osteopontin)	*Spp1*	↑	↑	++++
**Extracellular matrix constituents**				
***Basal lamina and ECM structural constituents***				
Agrin	*Agrn*	↓ *	↑ *	+++++ M
Biglycan	*Bgn*	↓ *	↑ *	+++++ M
Decorin	*Dcn*	↑ *	↑	++++
Elastin	*Eln*	↑ *	↑	+++++
Fibrillin 1	*Fbn1*	⇅	↑ *	+++++ M
Fibronectin 1	*Fn1*	↓ *	↑ *	+++
Fibulin 1	*Fbln1*	↓	↓	++++
Collagens	*Col1a1*	↑	↑	+++++
	*Col1a2*	↑	↑ *	+++++
	*Col3a1*	↑ *	↑ *	+++++
	*Col4a1*	⇅	↑ *	+++++ M
	*Col5a1*	↑	↑ *	+++++
	*Col5a2*	↑	↑ *	+++++
	*Col6a1*	↑	↑ *	+++++
	*Col6a3*	↑	↑ *	+++++
	*Col8a1*	↓	↑ *	+++++
	*Col9a3*	⇅	↑ *	+++++ M
	*Col14a1*	↑ *	↑ *	++++
	*Col16a1*	↑	↑ *	+++++
	*Col18a1*	↓	⇅	+++
Laminins	*Lama2*	↓ *	↑	+++++
	*Lama5*	⇅	↑ *	++ M
	*Lamb1*	⇅	↑ *	+++++ M
	*Lamb2*	↓ *	↑ *	++++ M
	*Lamc1*	↑ *	↑ *	++
	*Lamc2*	⇅	↑	+++++
Integrins	*Itga1*	↑	↓	++
	*Itga4*	↓	↑ *	++++ M
	*Itga5*	↓	↓	+++
	*Itga7*	↓	↓ *	+++
	*Itgb1*	↑	↑ *	++++
	*Itgb2*	↓ *	↑ *	+++++ M
	*Itgb4*	⇅	↑ *	+++++ M
	*Itgb6*	⇅	↑ *	+++++ M
	*Itgb7*	↑	↑ *	+++
Nidogens	*Nid1*	↑ *	↑ *	+++++ M
	*Nid2*	↓	↑ *	+++++ M
Secreted protein, acidic, cysteine-rich, (osteonectin, SPARC)	*Sparc*	↑ *	↑ *	+++++
***ECM proteases and inhibitors***				
Matrix metallopeptidases	*Mmp2*	↓	↓	++++
	*Mmp9*	⇅	⇅	+++
	*Mmp12*	↑	↑ *	+++++ M
	*Mmp14*	↓	↑ *	+++
	*Mmp16*	↑	↑	+++
	*Mmp19*	⇅	↑ *	+++
	*Mmp23*	↑	↑ *	++++ M
TIMP metallopeptidase inhibitor 1	*Timp1*	↑	↑ *	++
A disintegrin and metalloproteinase with thrombospondin motifs 1	*Adamts1*	↓	↑	+++++
Suppression of tumorigenicity 14 (matriptase)	*St14*	⇅	↑ *	+++++ M
***Other ECM associated molecules***				
Connective tissue growth factor	*Ctgf*	⇅	↑ *	+++++ M
Elastin microfibril interfacer 1	*Emilin1*	↑	↑ *	++++
Glypican 3	*Gpc3*	↓	↑ *	+++++ M
Periostin, osteoblast specific factor	*Postn*	↑	↑	+++++
Syndecans	*Sdc1*	↑ *	↑	++++
	*Sdc2*	↓ *	↓ *	+++
***Matriptase (St14) network components***				
Hepatocyte growth factor	*Hgf*	⇅	↑ *	+++
Hepatocyte growth factor activator	*Hgfac*	↓	↓	+++
Hepsin	*Hpn*	↓	↓ *	+++
Kallikrein B, plasma 1	*Klkb1*	↓	↓ *	++++
Plasminogen	*Plg*	↓ *	↓ *	+++
Plasminogen activator, urokinase	*Plau*	↑	↑ *	+++
Protease, serine, 1 (trypsin 1)	*Prss1*	⇅	↑	+++++
Protease, serine, 8 (prostasin)	*Prss8*	↑	↑	++++
Serine peptidase inhibitor, Kunitz type 1 (HAI-1)	*Spint1*	⇅	↑	+++++
Serine peptidase inhibitor, Kunitz type 2 (HAI-2)	*Spint2*	↓ *	↓ *	++
Serine peptidase inhibitor, clade A (α-1 antiproteinase), member 1	*Serpina1*	↓ *	↓	+++
Serine peptidase inhibitor, clade C (antithrombin), member 1	*Serpinc1*	↓	↓	+++++
Serine peptidase inhibitor, clade F (α-2 antiplasmin), member 1	*Serpinf2*	↓ *	↓ *	+++++

A hypothetical list of constituents involved in extracellular matrix remodeling based on existing literature (See text for details). A list of significantly up- or down-modulated transcripts is provided in Additional file 1 and a graphic illustration of signal intensities in Additional file 2. Transcriptional profile: ↓, down-modulated; ↑, up-modulated; ⇅, not modulated. Correlation strength: +++++, 0.90 to 1.00, very strong; ++++, 0.70 to 0.89, strong; +++, 0.40 to 0.69, modest; ++, 0.20 to 0.39 weak; + 0.00 to 0.19, very weak. M, transcript levels modulated mainly in the second tier of defense. *Significantly modulated transcripts (*P* < 0.01, false discovery rate (FDR) < 5%). The product–moment correlation coefficient (*r*) was calculated for signal intensities between transcripts of interest relative to *Krt1-19*, a marker of the hepatic duct/progenitor cells.

Second, to gain detailed information of the underlying molecular constituents in the remodeling ECM, when a HPC response is mounted, we used global transcriptional profiling to identify transcripts that are differentially expressed in a second tier of defense as compared with a first tier of defense. It is now agreed that the HPC response in a second tier of defense can be divided into several distinct phases [16]. In particular, these phases are well recognized in the AAF/PHx protocol of HPC-dependent liver regeneration in rat, where they can be distinguished on the basis of the morphological appearance of HPCs expressing the fetal liver proteins Afp and Dlk1 [17,18]. In this protocol, regeneration from HPCs is achieved through treatment with 2-acetylaminofluorene to block proliferation of existing hepatocytes followed by a growth stimulus provided by a 70% surgical hepatectomy (the AAF/PHx protocol). In the activation phase, a few proliferating HPCs can be detected in the biliary ductules and canals of Hering. These early HPCs express cytokeratin 19 (CK19) but not Dlk1 and Afp proteins. In the early proliferation and migration phase, multiple proliferating CK19-positive HPCs are detected, while cellular expression of Dlk1 and Afp proteins is rare. However, in the late proliferation and migration phase, an intricate network of tortuous structures of HPCs expressing CK19, as well as Dlk1 and Afp proteins, originates from the canals of Hering and expands into the hepatic parenchyma. This is in strong contrast with the cellular response mounted in a first tier of defense to liver injury, where existing hepatocytes and cholangiocytes are allowed to proliferate to reconstitute liver mass and function. In this study, we therefore performed transcriptional profiling on rat livers responding with a first-tier (PHx protocol) and a second-tier (AAF/PHx protocol) defense to injury. Our data analysis was focused on transcriptional changes in ECM constituents by a comparison of the transcriptional signature of untreated rat liver (control) with those from livers on day 1, day 5 and day 9 post hepatectomy in the two liver injury protocols. The time points were chosen to represent the activation phase (day 1), the early proliferation and migration phase (day 5), and the late proliferation and migration phase (day 9) of HPCs in a second tier of defense.

Third, using a recently established strategy for computerized three-dimensional analysis [19], we attempted to reconstruct and visualize the HPC response using the AAF/PHx protocol and Dlk1 as the immunohistochemical marker for HPCs, HAI-1 for epithelial cells in the biliary tree, and entactin/nidogen1 as a structural constituent of the ECM.

And fourth, to test the hypothesis that a selective difference in expression of ECM constituents would signify an activated HPC response, the tissue expression and localization of selected ECM constituents (that is, Col1a1, laminin, nidogen1, agrin, and integrin-β6) was analyzed, together with markers for the hepatic progenitor cell response (that is, Dlk1, HAI-1, OV6/CK19) by dual or triple fluorescence microscopy in different models of first and second tier defenses to liver injury. The presented data are expected to aid novel research in extracellular matrix biology and the field of hepatic stem cells, including a putative progenitor cell niche and its function in tissue regeneration and fibrogenesis after liver injury.

## Results

### Molecular composition of the extracellular matrix during a hepatic progenitor cell response

To deduce changes in the molecular composition of ECM during a HPC response we first created a hypothetical list of ECM constituents based on findings in the literature (see Table 1). We then used global transcriptional profiling to identify transcript levels of ECM constituents that change when a progenitor cell response is mounted in response to liver injury. We opted to study the changes in expression patterns in a second tier of defense, represented by the AAF/PHx protocol, and compare the changes to those observed in a first tier of defense, represented by the PHx protocol. A total of 4,653 and 3,416 transcripts were significantly up- or down-modulated in the AAF/PHx- and PHx protocols, respectively, at the nominal 0.01 level of the univariate test and employing the criteria of a false discovery rate (FDR) <5% and signal intensities >75 at days 1, 5 or 9 as compared to control. A decisive factor for choosing these criteria in the statistical analysis was detection of *Krt1-19*, *Afp*, *Tacstd1, Dlk1*, *Dmbt1*, *Gstp1*, *Gja1*, *Prom1*, *Cd24*, and *Vim* as differentially modulated transcripts because they have previously been shown to correlate with a HPC response during a second tier of defense in the AAF/PHx protocol [20]. In this study, their transcriptional profiles were consistent with a phasic HPC response, that is, low and high levels of transcripts at day 1 (activation phase) and day 9 (late proliferation and migration phase), respectively, while no changes were found after PHx. Importantly, 39 and 15 transcripts encoding ECM constituents were up- or down-modulated (*P* < 0.01, FDR <5%) at days 1, 5, or 9, as compared with controls in the AAF/PHx- and PHx protocols, respectively. Lists of significantly modulated transcripts representing ECM constituents in the two protocols, as well as a graphic illustration of signal intensities, are provided in additional files (see Additional files 1 and 2). A summary of the transcriptional profiles is presented in Table 1.

Interestingly, when we calculated the product–moment correlation coefficients (*r*) of *Krt1-19* signal intensities against the signal intensities of the transcripts related to the HPC response, very strong correlations were found (see Table 1 and panel a in Additional file 2). These findings prompted us to investigate further the transcriptional profiles of ECM constituents. As summarized in Table 1, the levels of numerous transcripts encoding different subunits of collagens (that is, *Col1a2*, *Col3a1*, *Col4a1*, *Col5a1*, *Col5a2*, *Col6a1*, *Col6a3*, *Col8a1*, *Col9a3*, *Col14a1*, *Col16a1*), laminins (that is, *Lamb1*, *Lamb2*), integrins (that is, *Itga4*, *Itgb2*, *Itgb4*, *Itgb6*), and various other ECM structural components (that is, *Agrn, Bgn, Fbn1, Nid1, Nid2, Sparc*) showed significant changes and strong or very strong correlations with *Krt1-19* and thus the HPC response in the second tier of defense. The levels of a few transcripts displayed significant changes but weak or modest correlations with *Krt1-19* (that is, *Fn1*, *Lama5*, *Lamc1*, *Itga7*). Furthermore, although the levels of a number of transcripts changed in both the first and second tiers of defense, a smaller number (that is, *Agrn*, *Bgn*, *Fbn1*, *Col4a1*, *Col9a3*, *Lama5*, *Lamb1*, *Lamb2*, *Itga4*, *Igtb2*, *Itgb4*, *Itgb6*, and *Nid1*) were significantly up-modulated, mainly in the second tier of defense. Taken together, our transcriptional profiling analysis suggests that, in a second tier of defense modulation of transcript levels of a number of ECM constituents are related to the HPC response. However, some are regulated in both first and second tiers of defense, while others are mainly modulated in the second tier of defense.

### A novel protease system with a potential role in ECM remodeling during a hepatic progenitor cell response

**Figure 1:**
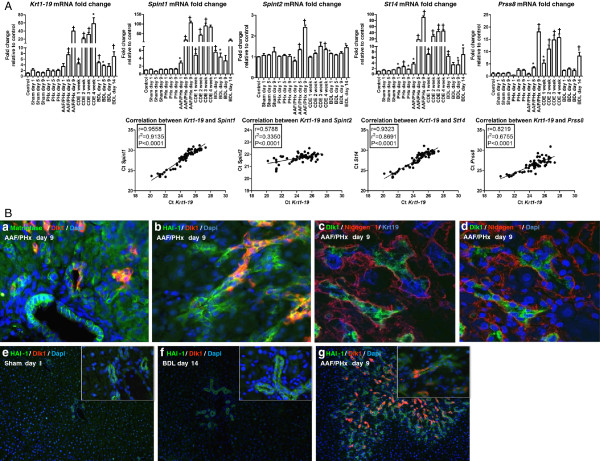
**Expression of matriptase network in hepatic injury. (A)** Real-time RT-PCR analysis. Elevated expression levels of *Spint1*, *St14*, *Prss8*, and *Krt1-19* transcripts were particularly evident in the two liver injury protocols with HPC-responses. While *Krt1-19* transcripts were significantly increased up to 7-fold on day 14 in the BDL protocol, HPC response showed the highest induction, with increases from 3- to 40-fold on days 1 through 9 in the AAF/PHx protocol and from 4- to 60-fold at weeks 1 through 4 in the CDE protocol. Similar expression patterns were obtained for *St14* and *Spint1* transcripts. Where *St14* was increased 2.6- to 7-fold in the PHx and BDL protocols, a 45- to 90-fold increase was observed on week 4 in the CDE and on day 9 in the AAF/PHx protocols, respectively. Similarly, *Spint1* reached a 90- to 105-fold increase on week 3 in the CDE and day 9 in AAF/PHx protocols, whereas *Prss8* increased 16- to 18-fold, respectively. In control and sham protocols, significant changes in gene expression were not detected. Values are relative to the uninjured control group ± standard deviation (*P < 0.05; †*P* < 0.001). Very strong positive correlations were observed between expression values for *Spint1* and *St14* and the progenitor cell marker *Krt1-19*. **(b)** Triple immunofluorescence localized: **(a)** matriptase, **(b)** HAI-1, and **(c)** OV6/Krt19 to both the cholangiocytes and HPC response. **(a-d)** Dlk1 marked a subpopulation of matriptase or HAI-1-positive cells in the HPC response. **(c,d)** Nidogen1 closely encircled the tortuous structures in the HPC response. **(e-g)** HAI-1 was expressed by cholangiocytes and HPCs regardless of type of hepatic insult. Dlk1, however, was only located to a subpopulation of cells in the HPC response. Magnification: 10×; inserts 40×.

Studies on the AAF/PHx protocol have shown that levels of matrix metalloproteinases (MMP) 2 and 9, which digest laminin and collagen IV, are increased in the HPC response [13]. In this study, our transcriptional profiling showed decreasing (*Mmp2*) or almost constant (*Mmp9*) levels over the period investigated (see Table 1 and panel h in Additional file 2). However, significant modulation of transcript levels related to the HPC response was found for *Mmp12*, *Mmp14*, *Mmp19*, and *Mmp23* but only *Mmp12* transcript levels showed a very strong correlation with that of *Krt1-19*. Interestingly, a previous microarray analysis using the AAF/PHx protocol in rats has also indicated elevated transcript levels of *St14* in HPCs [20]. *St14* encodes matriptase, a transmembrane serine protease with broad-spectrum degrading capabilities. It can activate the potent mitogen hepatocyte growth factor (HGF encoded by *Hgf*) [21]. Its substrates additionally encompass ECM constituents, including laminin, fibronectin, and collagen IV, as well as urokinase plasminogen activator, plasminogen, hepsin, and kallikrein B1 (encoded by *Plau*, *Plg*, *Hpn*, and *Klkb1*). Matriptase forms a functional unit with prostasin (encoded by *Prss8*) [22]. Two cognate inhibitors of matriptase, the transmembrane serine proteases HAI-1 and HAI-2 (encoded by *Spint1* and *Spint2*) exist. In addition to inhibiting matriptase activity, HAI-1 and HAI-2 localize matriptase to the cell surface, and inhibits HGF activity through inhibition of hepatocyte growth factor activator (encoded by *Hgfac*) [23-25]. By calculating product–moment correlation coefficients of the signal intensities for molecules in the matriptase system, we found a strong or very strong correlation between the up-modulated transcriptional profiles of *Krt1-19* and *St14*, *Spint1*, and *Prss8* and a weak correlation with the down-modulated profile of *Spint2* in the second tier of defense but only *St14* and *Spint2* were found to be significantly modulated (*P* < 0.01) in our statistical analysis (see Table 1, Additional file 1, and panel i in Additional file 2). Therefore, we investigated the transcriptional modulation of *Spint1*, *Spint2*, *St14*, *Prss8*, and *Krt1-19* (see Figure 1A), using real-time RT-PCR on RNA isolated from several rodent protocols of liver injury, that is: (a) a 70% hepatectomy (PHx) as an inducer of mature hepatocyte and cholangiocyte proliferation in a first tier of defense, (b) induction of HPC-responses in a second tier of defense using the AAF/PHx and choline-deficient, ethionine-supplemented (CDE) protocols and (c) induction of mature cholangiocyte proliferation using a bile duct ligation (BDL) protocol as a model of cholestatic liver disease and a first tier of defense. Two control groups, that is, untreated (control) rats and rats subjected to a sham laparotomy operation (sham) were included. Real-time RT-PCR analyses in the AAF/PHx protocol confirmed trends obtained from the microarray data. In fact, elevated expression levels of *Spint1*, *St14*, *Prss8*, and *Krt1-19* transcripts were particularly evident in the two second tier of defense protocols mounting HPC-responses. However, the analysis also showed significant up-modulation of *Spint1* transcript levels, mainly in the second tier of defense and a calculation of the product–moment correlation coefficient of Ct-values showed very strong positive correlation for *Krt1-19* versus both *Spint1* and *St14*. In conclusion, the real-time RT-PCR analysis confirmed the data displayed by the signal intensities in the microarray analysis. Our data therefore underscore a possible important role of the matriptase network in ECM remodeling, when a HPC response is mounted in a second tier of defense to liver injury.

### Computerized three-dimensional reconstruction of hepatic progenitor cell response using Dlk1, HAI-1 and nidogen1 as marker proteins for HPCs and ECM

Classical immunohistochemistry was employed to pinpoint the cellular localization of matriptase and HAI-1 (encoded by *St14* and *Spint1*, respectively; see Additional file 3). We studied livers from control, BDL day 14, AAF/PHx day 9, and CDE week 3. Both proteins were localized in membranes of cholangiocytes in the biliary tree of control liver and in the BDL protocol. In the AAF/PHx and CDE protocols, HAI-1 and matriptase additionally localized to membranes of the HPC response in the tortuous network of ductular structures radiating from the portal area into the parenchyma.

**Figure 2:**
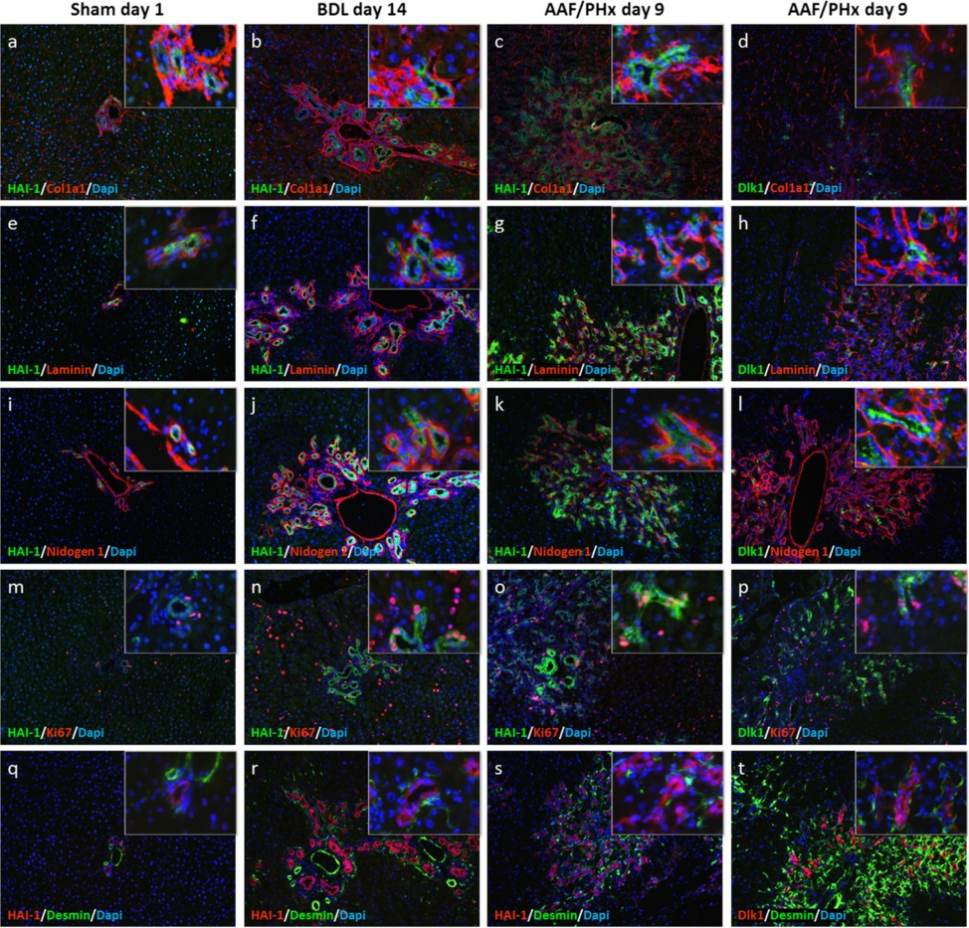
**Protein of the basal lamina and other ECM structural constituents are localized similarly across rat hepatic injury protocols.** In the sham, BDL, and AAF/PHx protocols, **(a-d)** Collagen1a1, **(e-h)** laminin, and **(i-l)** nidogen1 stained the portal vein endothelia and closely circumscribed cells in the biliary tree and HPC response. Collagen1a1 deposition was additionally observed in the parenchyma. In the canals of Hering, staining for HAI-1 often extended beyond the extracellular matrix (inserts in **c,g,j**). **(d,h,l)** The Dlk1 expressing subpopulation of cells in the AAF/PHx protocol was always in contact with the extracellular matrix. In the BDL and AAF/PHx protocols, **(n-p)** Ki67-positive cells were observed both within **(n,o)** the HAI-1- and **(p)** the Dlk1-positive cell populations. Ki67-positive cells were, however, rarely detected in **(m)** the sham protocol. In the sham protocol, **(q)** desmin prominently stained the portal vein endothelia and the portal artery, while faintly marking occasional star-shaped cells near the biliary tree or in the parenchyma. Regardless of injury type, in both the **(r)** BDL and **(s,t)** AAF/PHx protocols, numerous desmin-positive star-shaped cells closely escorted the biliary tree and HPC response.

To further characterize HAI-1 and matriptase expression in liver injury, double immunofluorescence on frozen liver sections from sham day 1, BDL day 14 and AAF/PHx day 9, were conducted relative to Dlk1, a marker of fetal hepatocyte-like HPCs. As a result of cross-reaction between secondary antibodies with primary sheep and goat antibodies, co-staining HAI-1 (goat) with matriptase (sheep) was not possible. The analysis revealed that HAI-1 and matriptase were expressed in the basolateral membranes of cholangiocytes of the biliary tree and HPCs. Interestingly, only a subpopulation of HAI-1-positive cells co-expressed Dlk1 (see Figure 1B, panels a, b, e, f, and g). A similar distribution of Dlk1-positive cells was found within the OV6/Krt19-positive HPC response (see Figure 1B, panels c and d). Dlk1-expressing HPCs were never detected within untreated or sham liver (see Figure 1B, panel e). Furthermore, Dlk1 expressing cells could not be detected in the biliary tree after a ligation of the common bile duct (see Figure 1B, panel f). In all injury protocols, as well as in untreated liver, nidogen1 staining was detected in the ECM on which cholangiocytes and HPCs were situated (see Figure 1B, panels c and d and Figure 2, panels i-l (inserts showing canals of Hering in high magnification)).

**Figure 3:**
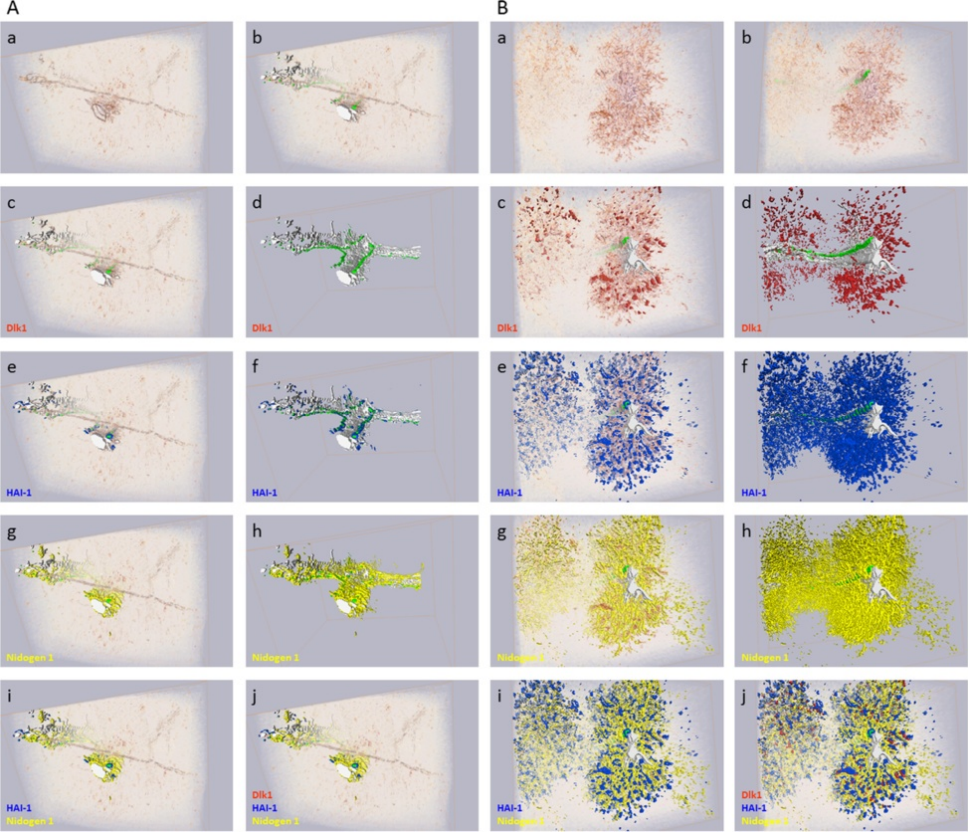
**Three-dimensional reconstruction of injured rat liver mounting a HPC response.** Three-dimensional (3D) reconstructions of 208 μm rat liver tissue demonstrated protein expression in control liver **(A)** and liver from day 9 in the AAF/PHx protocol **(B)**. Hepatic tissues were visualized by **(a)** volumetric rendering presenting the immunohistochemistry and **(d,f,h)** segmentation based reconstructions of lumina and protein expression. These techniques are presented combined in subpanels **b, c, e, g, i,** and **j**. In control rat liver **(A, a-j)** and day 9 in the AAF/PHx protocol **(B, a-j)** two portal areas merged in one end of the image stack **(d)** and continued as separate entities. Presented in green, lumina of slender portal bile ducts **(b)** along the portal vein connecting the portal areas could be traced in both livers. Whereas Dlk1 localized peripherally to the portal area in the AAF/PHx protocol **(B;c,d)** and was undetectable throughout the image stack from control liver **(A;c,d)**, HAI-1 marked the epithelial cells in the entire biliary tree **(A, e and f)** and the HPC response **(B;e,f)**. In control liver **(A;g,h)** Nidogen1 was only expressed in the portal area and circumscribed the biliary tree, and additionally embedded the HPC response in the AAF/PHx protocol **(B;g,h)**. Combined staining of HAI-1 and Nidogen1 emphasized nidogen1 deposition around the HAI-1, expressing cells in the bile epithelium **(A,B;i)**, and clarified Dlk1 as a marker of a subpopulation of HAI-1 expressing cells within the HPC response **(B;j)**.

It has previously been reported that Dlk1 is expressed in a subpopulation of the HPC response [18,26]. In an attempt to visualize the localization of Dlk1 expressing populations of fetal hepatocyte-like HPCs in a second tier of defense, we reconstructed the HPC response in three dimensions. Serial sections representing 208 μm of hepatic tissue from either untreated or AAF/PHx day 9 livers were stained by conventional immunohistochemistry for Dlk1, HAI-1, and nidogen1. Stained sections were translated to digital volumetric and segmentation based three-dimensional reconstructions (see Figure 3). Additional movie files show this in more detail (see Additional files 4 and 5). The reconstructions demonstrated that branching portal veins and accompanying portal bile ducts in one end of the stack directly coupled two portal areas that appeared distinct in following sections. In untreated as well AAF/PHx day 9 livers, nidogen1 closely circumscribed the portal vessels, bile ducts, and HPC response, whereas HAI-1 marked the two latter. Our computerized three-dimensional reconstruction visualized the intricate network of tortuous structures of HAI-1-positive HPCs embedded in an ECM microenvironment that expands into the hepatic parenchyma in the AAF/PHx protocol during a second tier of defense. Furthermore, in the AAF/PHx day 9 liver, Dlk1-positive HPCs located in smaller clusters at the portal area periphery within a HAI-1-positive HPC response. At no point throughout the image stacks did cells in the portal bile duct stain for Dlk1. Neither did any cell in the untreated liver express Dlk1. These data indicate that Dlk1 selectively marks a peripheral fetal hepatocyte-like subpopulation, whereas HAI-1 is expressed in cholangiocytes in normal liver as well as in the ductular epithelial cells of the entire HPC response.

### The molecular composition of ECM in different models of liver injury

Finally, we wanted to test the hypothesis that a selective difference in the molecular composition of the ECM would signify the activation of the HPC response in a second tier of defense. For this purpose, we conducted double immunofluorescence on liver sections from BDL day 14 (a model of a first tier of defense with respect to proliferation of cholangiocytes in the biliary tree) and AAF/PHx day 9 (as a model of the HPC response in a second tier of defense) and compared the results for those with uninjured liver from sham day 1. We investigated expression of the extracellular matrix molecules Collagen1a1, laminin, and nidogen1 in relation to HAI-1 and Dlk1 (see Figure 2) as well as integrin-β6 and agrin in relation to OV6/Krt19 (see Additional file 6). Further immunofluorescence characterization of differentially modulated ECM constituents, such as Lamb1, Col4a1, and Col5a1, was attempted but hampered by the availability of appropriate antibodies.

As illustrated in Figure 1B, panels b and e-g, immunofluorescence detection of HAI-1 and Dlk1 clearly revealed HAI-1 expression in the cholangiocytes of livers from sham and BDL protocols, whereas Dlk1 was undetectable. Similar results were found when integrin-β6 was combined with Dlk1 (see panels a and b in Additional file 6). However, whereas HAI-1 and integrin-β6 staining decorated cells in the entire intricate network of tortuous structures of HPCs as well as cholangiocytes in the large bile ducts in the AAF/PHx protocol, Dlk1 marked a peripheral subpopulation of fetal hepatocyte-like cells in the structures containing HPCs (see Figure 1B, panel g and panel d in Additional file 6). Double immunofluorescence analysis of HAI-1 with Collagen1a1 (see Figure 2, panels a-c), laminins (see Figure 2, panels e-g), and nidogen1 (see Figure 2, panels i-k) demonstrated ECM deposition around portal area vessels, the biliary tree and the tortuous structures of HPCs. Collagen1a1 additionally stained the portal mesenchyma and was found scattered along the sinusoids. Double immunofluorescence of the ECM structural constituents with Dlk1 in the AAF/PHx protocol depicted the HPC response as ductular structures, some, but not all, harboring clusters of Dlk1 expressing HPCs (see Figure 2, panels d, h, and l).

The intermediate filament desmin marked the endothelia in the portal area vessels and few cells scattered in the parenchyma (see Figure 2, panels q-t). However, in both the BDL and AAF/PHx protocols, numerous star-shaped desmin-positive cells were closely associated with the cholangiocytes in the biliary tree and the HPC response. In sham day 1 liver, the marker Ki67 demonstrated that few cells in the parenchyma and biliary tree were proliferating. In the BDL and AAF/PHx protocols, multiple Ki67-positive cells were detected in the HAI-1-positive cholangiocytes of the biliary tree and the tortuous structures of HPCs (see Figure 2, panels m-p).

## Discussion

When liver regeneration through hepatocyte division is arrested, dormant progenitor cells in the canals of Hering are activated and give rise to intricate ductular structures radiating into the parenchyma, in what has been termed the second tier of defense to liver injury. These structures contain proliferating HPCs capable of differentiating into cholangiocytes or hepatocytes and are termed the HPC response [1-6]. Little is known of the exact composition of the microenvironment governing this form of regeneration, but several cellular and molecular players have been identified. These include stellate cells, myofibroblasts, and Kuppfer cells, as well as cytokines and growth factors [9,27,28].

Previous studies support the proposition of an important role for the ECM in establishing specific hepatic microenvironments or niches to modulate the response of HPCs to tissue injury [6,13,15,29]. However, a detailed scheme of the molecules involved in remodeling of the ECM when the hepatic progenitor cell niche responds to liver injury has not yet been established. The ECM is a dynamic scaffold influencing several aspects of cellular behavior, including growth, survival, and morphology, partly through the integrin family of ECM receptors [30,31]. Studies in different tissues across species have clarified that the ECM constitutes an important part of progenitor cell niches [7,8]. While certain microenvironments in *Drosophila*, as expected, repress stem cell differentiation and promote adherence to the niche [8], even age-related differences in ECM composition directly influence stem cell function [32]. To begin understanding the remodeling events in the ECM microenvironment when HPCs are activated in a second tier of defense to liver injury, we used the AAF/PHx protocol to study transcriptional changes in ECM constituents during three phases of an HPC response, that is, the activation phase, the early proliferation and migration phase, and the late proliferation and migration phase. In the HPC response, the levels of numerous transcripts encoding different subunits of collagens (that is, *Col1a2*, *Col3a1*, *Col4a1*, *Col5a1*, *Col5a2*, *Col6a1*, *Col6a3*, *Col8a1*, *Col9a3*, *Col14a1*, *Col16a1*), laminins (that is, *Lamb1*, *Lamb2*), integrins (that is, *Itga4*, *Itgb2*, *Itgb4*, *Itgb6*), and various other ECM structural components (that is, *Agrn, Bgn, Fbn1, Nid1, Sparc*) showed significant changes and strong or very strong correlations with the HPC-marker *Krt1-19.* The levels of a few transcripts displayed significant changes but weak or modest correlations with *Krt1-19* (that is, *Fn1*, *Lama5*, *Lamc1*, *Itga7*). Additionally, although the levels of a number of these transcripts changed in both the first and second tier of defense to liver injury, *Agrn, Bgn, Fbn1, Col4a1, Col9a3, Lama5, Lamb1, Lamb2, Itga4, Igtb2, Itgb4, Itgb6, and Nid1* were significantly up-modulated, mainly in the latter. We conclude that a number of basal lamina and ECM structural constituents are modulated at the transcriptional level to aid the evolution of a second-tier HPC response to liver injury.

Two of the modulated structural constituents, laminin and Collagen1, are well described in hepatic studies. Where laminin is a recognized marker of the basal lamina [33], the fibrillar Collagen1 heterotrimer is the main component of scarring tissue in cirrhotic liver [34]. Nidogen1 and agrin represent a glycoprotein [35] and a proteoglycan [36,37], respectively, both known to be important for ECM stabilization through their cross-linking abilities. In our study, laminin and Collagen1 circumscribed the portal area blood vessels and the cholangiocytes in the biliary tree in normal liver. Collagen1-positive stretches were additionally observed in the parenchyma, in what might be the space of Disse [33]. In the BDL protocol, where mature cholangiocyte proliferation models cholestatic liver disease, and the AAF/PHx protocol of HPC-driven regeneration, we observed laminin and Collagen1 deposition around the biliary tree and the intricate network of tortuous structures of HPCs. In the HPC response, combined detection of Dlk1 and laminin or Collagen1 consistently showed a subpopulation of Dlk1-positive cells located in duct-like stretches of ECM. The similarity of these Dlk1-positive HPCs with fetal hepatocytes might indicate the presence of a cellular hierarchy in the HPC response. Therefore, this and previous studies document laminin [2,3,6] and Collagen1 [15] deposition around HPCs, suggesting an important role for these components in the HPC response. Interestingly, in *in vitro* studies, opposed roles for laminin and Collagen1 with respect to HPCs have been observed. While laminin supports proliferation and expansion, Collagen1 elicits growth arrest and differentiation [38,39]. Moreover, a recent study indicated the requirement for α1-containing laminin in committing HPCs towards the cholangiocytic fate and establishing apico-basal polarization, whereas α5-containing laminin played a major role in mature bile duct formation [40]. In our studies, only *Lama5* transcriptional levels were changed significantly when a HPC response was stimulated. However, a significant transcriptional modulation was found for *Lamb1*, *Lamb2*, and *Lambc1*. The sequential and different actions of even structurally similar laminins, possibly mediated through integrin β1-signaling, emphasize the importance of our study deciphering the time and spatial changes of the actual molecular constituents of the ECM during a HPC response. Future studies directed towards the identified ECM constituents using cell lines *in vitro* as well as hepatic injury models with transgenic animals will be needed to elucidate further the microenvironment governing an HPC response.

Our transcriptional profiling also revealed that transcripts related to a novel protease system with functions in ECM remodeling and composed of matriptase (*St14*) and its associated protein HAI-1 (*Spint1*) were significantly elevated in a HPC response. Matriptase and HAI-1 are relatively undescribed in the liver. Previous studies have localized matriptase and HAI-1 to the bile duct epithelium and ductular reactions [41,42], while microarray analyses have indicated elevated mRNA levels of *St14* in the HPC response [20]. Moreover, down-regulation of HAI-1 and HAI-2 (encoded by *Spint2*) has recently been associated with differentiation of hepatoblast-derived cells into hepatocytes and cholangiocytes [42]. In our study, HAI-1 and matriptase not only stained cholangiocytes in the biliary tree of adult control liver but also the proliferating cholangiocytes in the BDL protocol and the entire intricate network of tortuous structures of HPCs in the AAF/PHx protocol. Dlk1 is a rare surface marker of HPCs associated with less differentiated hepatocellular phenotypes [17,18]. Co-staining with HAI-1 demonstrated that Dlk1 strikingly marked a subpopulation of HAI-1-positive epithelial cells only present in the HPC response. However, examining single sections of liver tissue could mask underlying connections not immediately apparent. We therefore applied three-dimensional reconstructions to visualize 208 μm of hepatic tissue from control and AAF/PHx treated rats. The reconstructions in the AAF/PHx protocol illustrated that while two portal areas connected within the image stack, the Dlk1/HAI-1-positive cells were entirely located in peripheral ductular structures connected to the larger bile ducts by HAI-1-positive cells. We therefore hypothesize that the AAF/PHx protocol gives rise to a subpopulation of HAI-1 and Dlk1-expressing transit-amplifying cells that can differentiate along the hepatocytic or cholangiocytic lineages in a sequence similar to that which has been observed during liver development. This suggests the presence of a cellular hierarchy in the activated progenitor cell niche.

Assuming that the niche for HPCs is localized in canals of Hering, it appears to be sharply limited by the deposition of Collagen1, laminin, nidogen1 and agrin, facilitating HPC adhesion, transit-amplification and early cholangiocytic or hepatocellular differentiation reflected by expression of HAI-1 alone (biliary) or HAI-1 and Dlk1 in combination (hepatobiliary), a phenomenon similar to that observed in *Drosophila* gonads [8]. Given that the same ECM proteins are also deposited around the biliary tree in the liver of untreated rats, the BDL protocol as well as in the HPC response in the AAF/PHx protocol, it seems conceivable that a main function of the investigated ECM proteins is to serve a supportive role for the biliary and hepatobiliary phenotypes, rather than to induce differentiation.

## Conclusions

This study presents a number of ECM constituents that are modulated at the transcriptional levels when a HPC response is mounted in a second tier of defense to liver injury. However, significant remodeling of extracellular matrix components also occurs at the post-translational level, involving protein processing by proteolysis. Furthermore, a number of different cell types apart from the biliary cells and HPCs, such as hepatocytes, stellate cells and myofibroblasts, are involved in the synthesis and processing of ECM constituents. Future studies using novel cell-sorting strategies and assays pinpointing post-translational modifications during ECM remodeling in the different animal models are needed to fully understand the functional roles of the identified ECM constituents in mounting and supporting a HPC response.

## Methods

### Animal protocols

Male Fischer (F344) rats (6 to 8 weeks, Taconic Europe A/S (Ry, Denmark)) were kept under standardized conditions with access to food and water *ad libitum*. Liver injury was induced by: (1) The PHx protocol, modeling a first tier of defense, where surgical resection of the median and left lateral liver lobes removed 70% of the liver mass and resulted in proliferation of mature hepatocytes and cholangiocytes; (2) the AAF/PHx protocol or a choline-deficient, ethionine-supplemented (CDE-protocol) diet, both modeling a HPC response in a second tier of defense. In brief, rats were treated with 9 mg/kg/day 2-acetylaminofluorene by gavage once daily for 9 days, interrupted at day 5 to perform a 70% hepatectomy, or fed a choline-deficient diet (ICN, Costa Mesa, CA) supplemented with 0.165% DL-ethionine (Sigma-Aldrich) in the drinking water; (3) the BDL protocol, a model of cholestatic liver disease and a first tier of defense, where the common bile duct was tied with a non-absorbable ligature, resulting in a ductular reaction of proliferating mature cholangiocytes; and (4) control groups, comprising: (a) a sham operation with laparotomy only (sham) and (b) untreated animals (control). Groups of three to six animals were sacrificed by cervical dislocation at the time points indicated. Studies were approved by The Danish Council for Supervision with Experimental Animals (License Nr. 2004/561-843 and 2009/561-1632).

### Transcriptional profiling

#### ***RNA isolation***

Liver samples were homogenized by means of a TissueLyser (Qiagen, Retsch, Germany) and total RNA was extracted using the Trizol Reagent (Invitrogen, Carlsbad, CA USA). The RNA concentration was measured using a NanoDrop ND-1000 spectrophotometer (NanoDrop Technologies, Inc., Wilmington, DE USA). The purity of extracted RNA was determined by the A260/A280 ratio, and a ratio of 1.8 to 2.0 was considered acceptable. The integrity of the RNA was confirmed on an Agilent 2100 Bioanalyzer and its corresponding Lab-on-a-chip product ‘Agilent RNA 6000 Nano Kit’ (Agilent Technologies Inc., Santa Clara, CA USA).

#### ***Illumina bead chip array analysis***

Biotin-labeled cRNA was produced by linear amplification (AMIL1791; Ambion, Austin, TX USA) using 200 ng of quality-checked total RNA. Liver samples obtained from rats treated according to the PHx or AAF/PHx protocols at days 1, 5, and 9 post hepatectomy as well as from untreated controls were prepared in triplicate from individual rats. Chip hybridizations, washing, Cy3-streptavidin (Amersham Biosciences, GE Healthcare) labeling, and scanning were performed on an Illumina BeadStation 500 (Illumina, San Diego, CA USA) platform using reagents and following protocols supplied by the manufacturer. cRNA (750 ng/sample) were hybridized on Sentrix whole genome bead chips RatRef-12 covering >22,000 RefSeq transcripts. Image analysis and data extraction were performed automatically using Illumina BeadScan Software 3.2 and the data set normalized using quantile and background subtraction. The analysis of differentially modulated genes was performed using BRB-ArrayTools Version 4.3.1 developed by Dr. Richard Simon and the BRB-ArrayTools Development Team. Genes showing signal intensities over 75 were defined to be differentially modulated if they were up- or down-regulated with a *P* value below 0.01 (Students *t* test) and a false discovery rate (FDR) < 5% when comparing control vs. PHx rats at days 1, 5, and 9 or AAF/PHx rats at days 1, 5, and 9.

#### ***Real-time RT-PCR***

cDNA synthesis was performed from 1 μg RNA per sample using a high-capacity cDNA reverse transcription kit with RNase inhibitor (Applied Biosystems, Foster City CA, USA). The probe and primer design (see Table 2), optimizations, PCR efficiency and real-time RT-PCR were executed on an ABI 7300 real-time PCR system (Applied Biosystems). Genes were quantified separately in triplicates using TaqMan® 2x Universal PCR Master Mix, No AmpErase® UNG (Applied Biosystems). As transcription of classical housekeeping genes have been shown to be modulated in liver injury models, which was also evident from our transcriptional profiling analysis, we opted to use equal amounts of reverse-transcribed quality-controlled RNA from individual animals in the real-time PCR analysis [43,44]. Results were obtained as threshold cycle values. Expression levels of target genes in the sample groups relative to the control group were calculated as fold changes.

**Table 2:** Applied primers and probes for real-time RT-PCR

**Gene symbol**	**Sequence**	**Threshold/concentration**
***Krt19***		0.052
^1^Primer forward	5′-GGC GCC ACC ATT GAG AACT-3′	100 nM
^1^Primer reverse	5′-GCC AGG CGG GCA TTG-3′	100 nM
^1^Probe	5′-FAM-CAA GAT AGT CCT ACA GAT CG-BHQ-1-3′	200 nM
***Spint1***		0.1
^1^Primer forward	5′-GCA TCT ACT GCA AGG TGA CCA A-3′	300 nM
^1^Primer reverse	5′-CCA CAG CTC AAC TTC TTC TGG AT-3′	300 nM
^2^Probe	5′-FAM-TGT CAG TGT TAA GAG AAA C-MGBNFQ-3′	280 nM
***Spint2***		0.1
^1^Primer forward	5′-TGC TCT GGG AAG CAG ATG TAT C-3′	600 nM
^1^Primer reverse	5′-TGC GAA CCA TTC GGA TCA G-3′	600 nM
^2^Probe	5′-FAM-ACA AAA GCG GTG ATC C-MGBNFQ-3′	100 nM
***St14***		0.1
^1^Primer forward	5′-TGG CTG TGG AGC GAG TTG T-3′	700 nM
^1^Primer reverse	5′-TGC CAC GGG CAT GGA-3′	700 nM
^1^Probe	5′-FAM-CCT TCC CCA TTG ACC CCA GAA TGC-BHQ-1-3′	240 nM
***Prss8***		0.1
^1^Primer forward	5′-CAG TCA AGA ATC GGA GCT GAT G-3′	300 nM
^1^Primer reverse	5′-CAC TGC CAC CAC CTG TGA TG-3′	300 nM
^1^Probe	5′-FAM-CTG AAG CTT CCT GTG GTG CAG TCA TCC-BHQ-1-3′	140 nM

Primer Express® Software Version 3 (Applied Biosystems) was employed to design primers within different exons and probes covering exon-exon borders to prevent amplification of genomic DNA. ^1^TAG Copenhagen, Copenhagen, Denmark; ^2^Applied Biosystems, Foster City, CA USA.

#### ***Statistical analysis***

Student’s *t* test in Microsoft Excel (http://www.microsoft.com/) was used for comparisons of affected liver with control liver. GraphPad Prism 5 (http://www.graphpad.com/) was used to calculate the product–moment correlation coefficient *r* and the coefficient of determination *r*^*2*^ between transcript threshold cycle values.

### Morphological analyses

#### ***Immunohistochemistry***

Immunohistochemistry was performed on 4 μm formalin-fixed paraffin-embedded liver sections (see Table 3). Antigen retrieval was used as necessary and sections incubated with primary antibodies overnight at 4°C. Primary antibody binding was visualized by means of appropriate Vectastain Elite ABC reagent kits (Vector laboratories, Inc. Burlingame, CA USA) using diaminobenzidine (Sigma-Aldrich, Saint Louis, MO USA) as substrate chromogen and counterstained with Mayer’s hematoxylin.

**Table 3:** Applied antibodies for immunohistochemistry and immunofluorescence

**Primary antibody**	**Dilution**	**Catalog number (clone)**	**Secondary antibody**	**Dilution**	**Catalog number**
**Immunohistochemistry**					
^1,^*Matriptase	1:500	AF3946	^7^Vectastain Elite ABC	Not applicable	PK-6106
^1,^*HAI-1	1:500	AF1141	^7^Vectastain Elite ABC	Not applicable	PK-6105
^2,+^Nidogen-1	1:1000	ab14511	^4^Dako EnVision + System-HRP	Not applicable	K4008
^3^DLK1	1:50	N/A	^4^Dako EnVision + System-HRP	Not applicable	K4008
**Immunofluorescence**					
^1^Integrin-β6	1:50	AF2389	^8^Alexa Fluor 488/594	1:400	A-11055/A-11058
^1^Agrin	1:100	AF550	^8^Alexa Fluor 488/594	1:400	A-11055/A-11058
^1^h/rOV-6	1:50	MAB2020 (Clone OV-6)	^9^Fluorescein	1:200	715-095-151
^1^HAI-1	1:50	AF1141	^8^Alexa Fluor 488/594	1:400	A-11055/A-11058
^1^DLK1	1:100	MAB114 (211309)	^8^Alexa Fluor 488	1:400	A-21141
^3^DLK1	1:250	N/A	^8^Alexa Fluor 594	1:400	A-21207
^4^Desmin	1:500	M0760 (D33)	^9^Fluorescein	1:200	715-096-151
^4^Laminin	1:2000	Z0097	^8^Alexa Fluor 594	1:400	A-11037/A-21207
^5^COL1A1	1:100	AB755p	^8^Alexa Fluor 594	1:400	A-11037/A-21207
^2^Nidogen-1	1:1500	ab14511	^8^Alexa Fluor 594	1:400	A-11037/A-21207
^6^Ki67	1:2000	NCL-Ki67p	^8^Alexa Fluor 594	1:400	A-11037/A-21207

^1^R&D Systems, Inc. Minneapolis, MN USA; ^2^AbCAM, Cambridge, UK; ^3^Polyclonal rabbit antibody; ^4^Dako A/S, Glostrup, Denmark; ^5^Milipore, Billerica, MA USA; ^6^Leica Microsystems Inc., Buffalo Grove, IL USA; ^7^Vector laboratories, Burlingame, CA USA; ^8^Life Technologies Europe BV, Nærum, Denmark; ^9^Jackson ImmunoResearch Europe Ltd., Suffolk, UK; *Sections were boiled in retrieval solution (St14) or retrieval solution, pH9 (Spint1) (Dako, catalog number S2367/S1699) for 20 min in a microwave oven and let to cool to room temperature for 20 min. ^+^Sections were digested in proteinase K for 2.5 minutes at room temperature (Dako, catalog number S3020).

#### ***Immunofluorescence***

For immunofluorescence analysis, 8 μm cryosections were fixed in 4% paraformaldehyde in phosphate buffered saline and incubated with primary antibodies overnight at 4°C (see Table 3). Primary antibody binding was visualized using appropriate Alexa Fluor (Invitrogen) or Fluorescein (Jackson ImmunoResearch Europe Ltd., Suffolk, UK) conjugated antibodies and sections were mounted with Prolong Gold anti-fade reagent with Dapi (Invitrogen).

#### ***Computer-assisted three-dimensional reconstructions***

Reconstruction of protein expression in rat liver in three dimensions was carried out using two previously developed protocols [19]. Briefly, 52 consecutive 4 μm thick sections were cut from formalin-fixed paraffin-embedded liver, representing a total length of 208 μm hepatic tissue. Every third section was stained by immunohistochemistry for Dlk1, HAI-1 or nidogen1 (see Table 3). Immunostained sections were digitized in tagged image file format (.tiff) at 10× magnification (894 × 668 μm) using a Leica DC300 FX CCD camera (24-bit red-green-blue color depths, 1392 × 1040 pixels) attached to a Leica DM4000 B microscope. Protein expression in the stack of aligned images was visualized in the software platform ‘Amira’ version 5.3.3 (http://www.amira.com) using two approaches; volume and segmentation based surface rendering, respectively.

## Abbreviations

AAF/PHx: 2-acetylaminofluorene (AAF) combined with 70% partial hepatectomy; BDL: Bile duct ligation; CDE: Choline-deficient, ethionine-supplemented diet; CK19: Cytokeratin 19; ECM: Extracellular matrix; FDR: False discovery rate; HAI: Hepatocyte growth factor activator inhibitor; HPC: Hepatic progenitor cell; MMP: Matrix metalloproteinase; PHx: 70% partial hepatectomy; RT-PCR: Reverse transcriptase polymerase chain reaction; SPARC: Secreted protein, acidic, cysteine-rich; TIMP: Tissue inhibitor of metalloproteinase.

## Competing interests

The authors declare that they have no competing interests.

## Authors’ contributions

HCB, PSV, and SST conceived the study. PJ and HCB designed and executed the animal studies. JBA, MR, SST, PSV, and HCB performed and analyzed the transcriptional profiles. PSV, TJ, JB, and LKV optimized and performed the qRT-PCR analyses. PSV, TATT, LMG, and CHJ optimized and performed the immunohistochemistry and immunofluorescence experiments. PSV developed and performed the computer-assisted three-dimensional reconstructions. All authors contributed to the writing of the paper and approved the final version.

## Supplementary Material

Additional file 1 **Table of genes encoding ECM constituents differentially expressed among classes in a first tier or second tier of defense using the PHx and AAF/PHx protocols as model systems.** The analysis of differentially modulated genes was performed using BRB-ArrayTools Version 4.3.1 developed by Dr. Richard Simon and the BRB-ArrayTools Development Team. Genes showing signal intensities over 75 were defined to be differentially modulated if they were up- or down-regulated with a *P* value below 0.01 (Students *t* test) and a false discovery rate (FDR) < 5% when comparing control vs. PHx rats at days 1, 5, and 9 or AAF/PHx rats at days 1, 5, and 9. Click here for file

Additional file 2 **Graphic illustration of signal intensities for genes encoding ECM constituents differentially expressed among classes in a first tier or second tier of defense using the PHx and AAF/PHx protocols as model systems.** Presentations of selected gene expression data from livers of control rats and rats subjected to the 2-acetylaminofluorene/70% partial hepatectomy protocol of progenitor cell activation. Transcripts are categorized according to function: (a) epithelial (oval) cell markers; (b) mesenchymal cell markers; (c,d) auxiliary proteins; (e) collagens; (f) laminins; (g) integrins; (h) matrix metallo peptidases and inhibitors; (i) and matriptase network components. Linear regressions and correlation values were calculated between signal intensities for transcripts versus the hepatic progenitor cell marker Krt19 (*Krt19)* or the mesenchymal cell marker desmin (*Des*). Transcripts with signal intensities below 75 were disregarded. Data are presented as ‘signal intensity’ ± standard error of the mean). Click here for file

Additional file 3 **Matriptase and HAI-1 highlights the cholangiocytic or biliary lineage.** Photomicrographs showing expression of (a) matriptase and (e) its cognate inhibitor HAI-1 in the epithelial cells of the biliary tree in control liver. Correspondingly, (b-d) matriptase and (f-h) HAI-1 mark the epithelial cells in the biliary tree of (b,f) bile duct ligated rat liver and the HPC response in two protocols of hepatic progenitor cell activation; (c,g) the 2-acetylaminofluorene/70% partial hepatectomy and (d,h) choline-supplemented ethionine-deficient diet, respectively. Magnification 40×. Click here for file

Additional file 4 **3D-reconstruction of a portal area from control rat liver.** Rotating volumetric rendering depicting aligned immunohistochemical staining for (A) HAI-1, Dlk1 and nidogen1. (B-D) Rotating segmentation based reconstructions of portal vessel lumina and protein expression. Click here for file

Additional file 5 **3D-reconstruction of a portal area on day 9 in 2-acetylaminofluorene/70% partial hepatectomized liver.** Rotating volumetric rendering depicting aligned immunohistochemical staining for (A) HAI-1, Dlk1 and nidogen1. (B-D) Rotating segmentation based reconstructions of portal vessel lumina and protein expression. Click here for file

Additional file 6 **Expression of investigated proteins is similar across rat hepatic injury protocols.** (a-d) Integrin-β6 and (a,b) OV6/Krt19 mark epithelial cells in the biliary tree in (a) sham operated and (b) bile duct ligated (BDL) rats, respectively. (c,d) Similar expression for integrin-β6 is observed in the 2-acetylaminofluorene/70% partial hepatectomy (AAF/PHx) protocol with a hepatic progenitor cell response. (e) Agrin highlights the portal vein endothelia, portal artery and encloses the biliary tree in sham operated liver. In both (f) the BDL and (g) AAF/PHx- protocols, agrin escorts OV-6/Krt19-positive cells. (inserts in f,g). OV6/Krt19- or Integrin-β6-positive terminal cells in the canal of Hering penetrating into the lobules frequently extend beyond deposited agrin. (h) Dlk1-positive subpopulations in HPC response are enclosed by agrin, (insert in h) the latter forming tubular structures depicting the extent of the ductular reactions. Magnification 10×, inserts 40×. Click here for file
