# Supplementary material for: Molecular constituents of the extracellular matrix in rat liver mounting a hepatic progenitor cell response for tissue repair
Source: Fibrogenesis Tissue Repair. 2013 Dec 20;6:21. doi: 10.1186/1755-1536-6-21 (PMC3892118; doi:10.1186/1755-1536-6-21)

Additional File 2 Panel a

Epithelial (oval) cell markers ± SEM

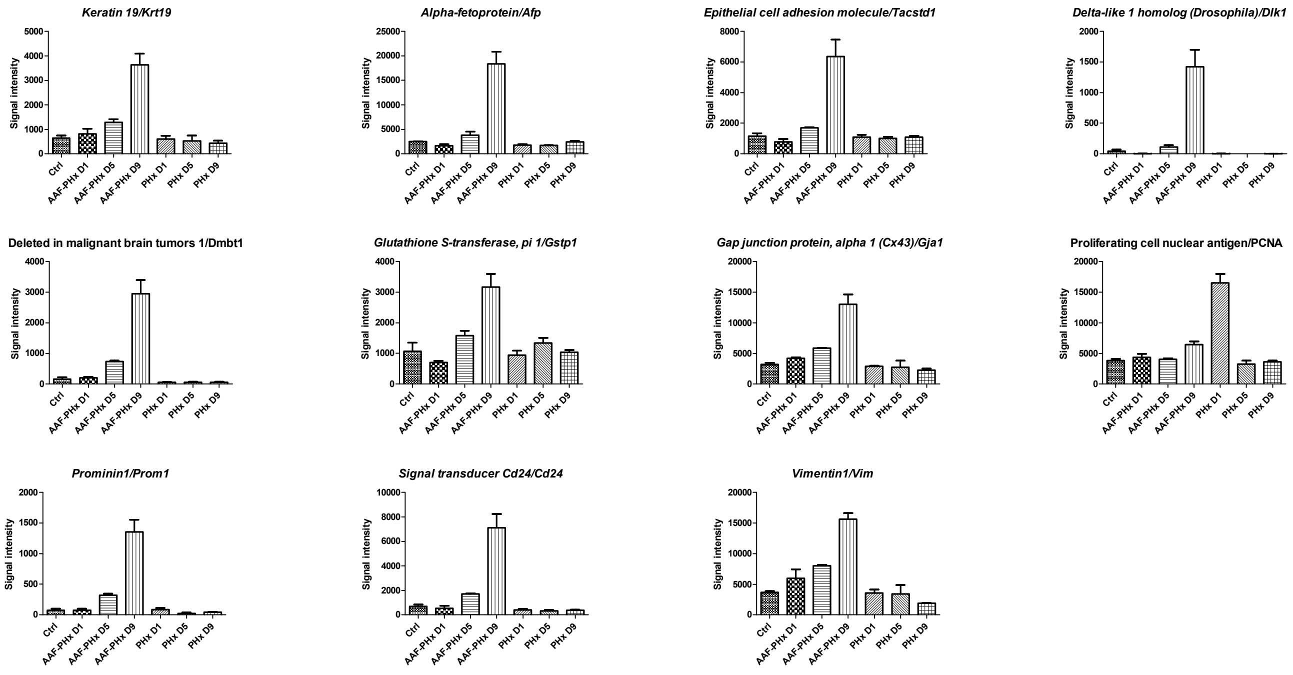

Krt19 versus epithelial (oval) cell markers

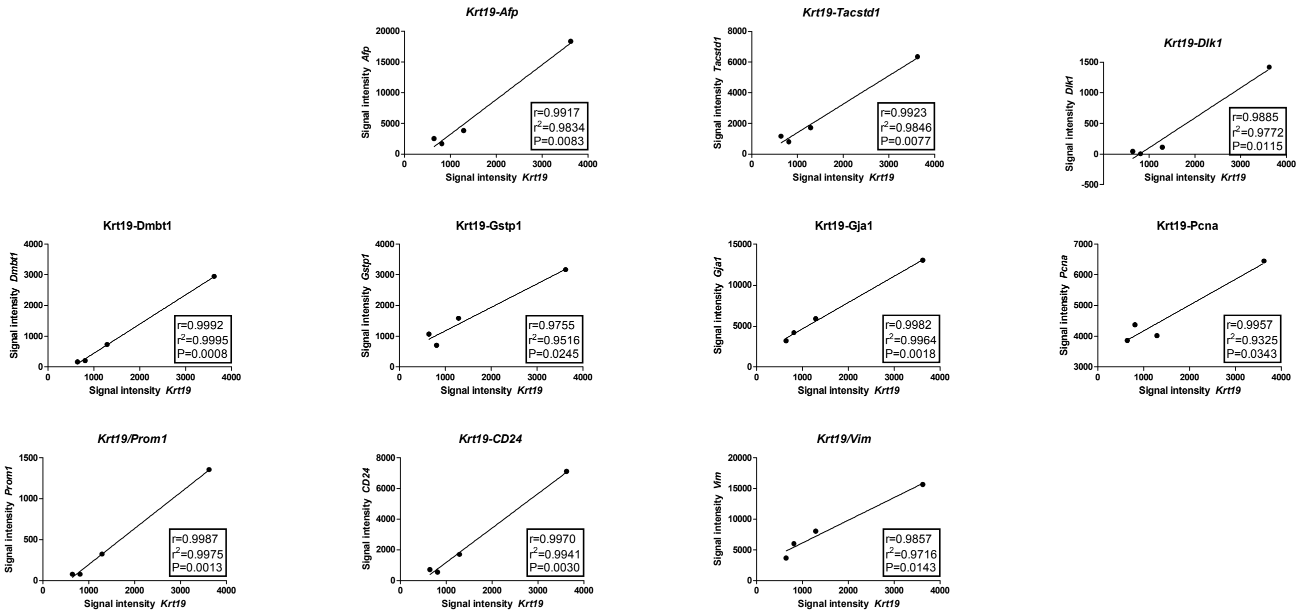

Des versus epithelial (oval) cell markers

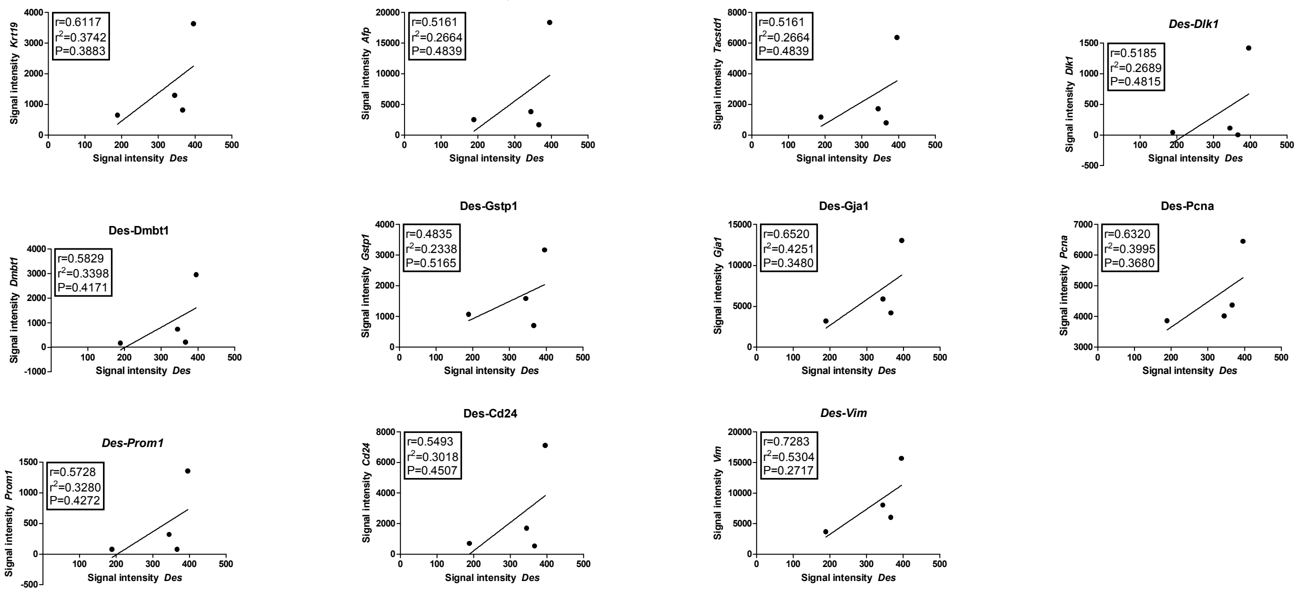

Additional File 2 Panel b

Mesenchymal cell markers ± SEM

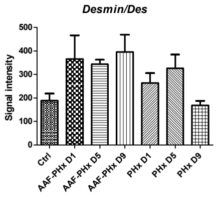

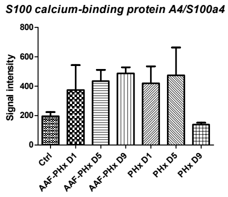

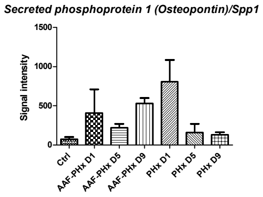

Krt19 versus mesenchymal cell markers

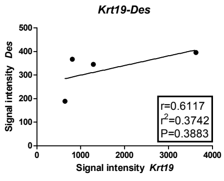

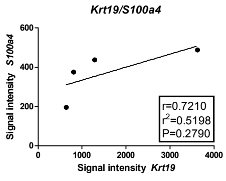

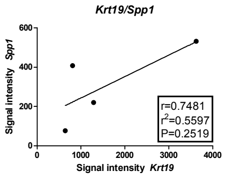

Des versus mesenchymal cell markers

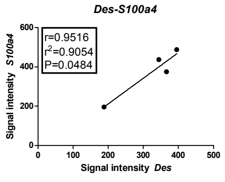

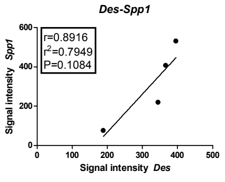

Additional File 2 Panel c

Auxiliary proteins ± SEM

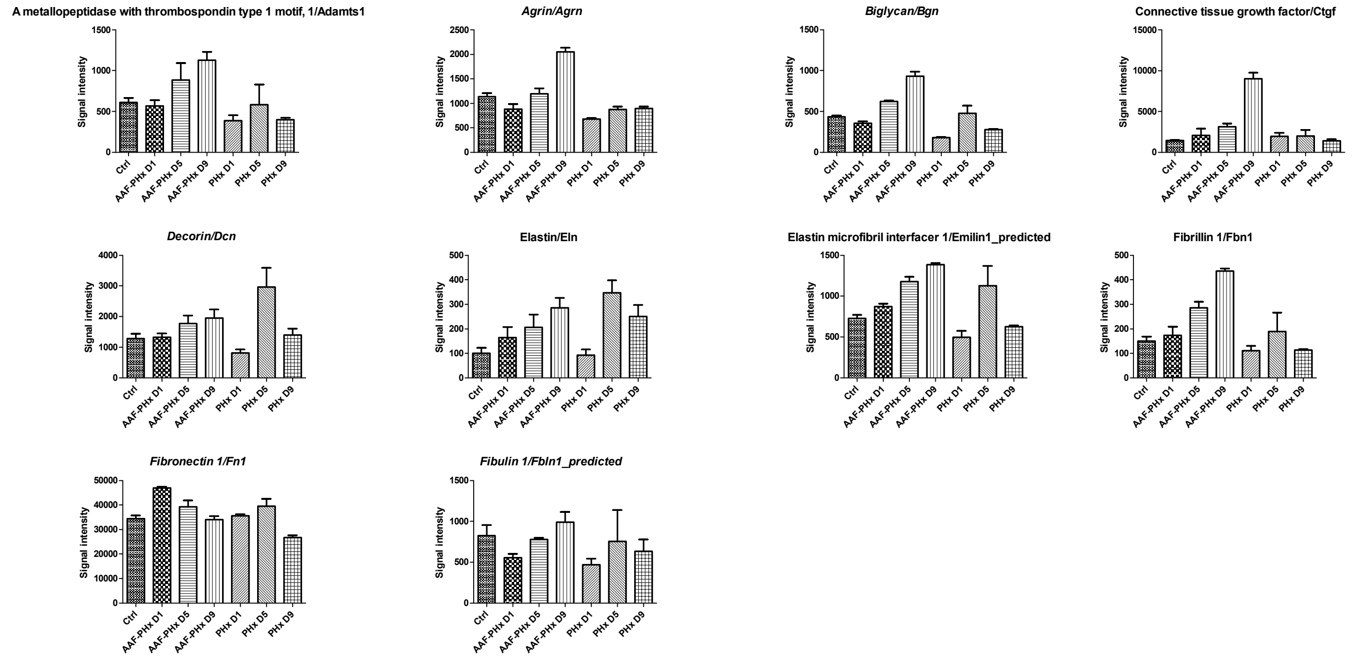

Krt19 versus auxiliary proteins

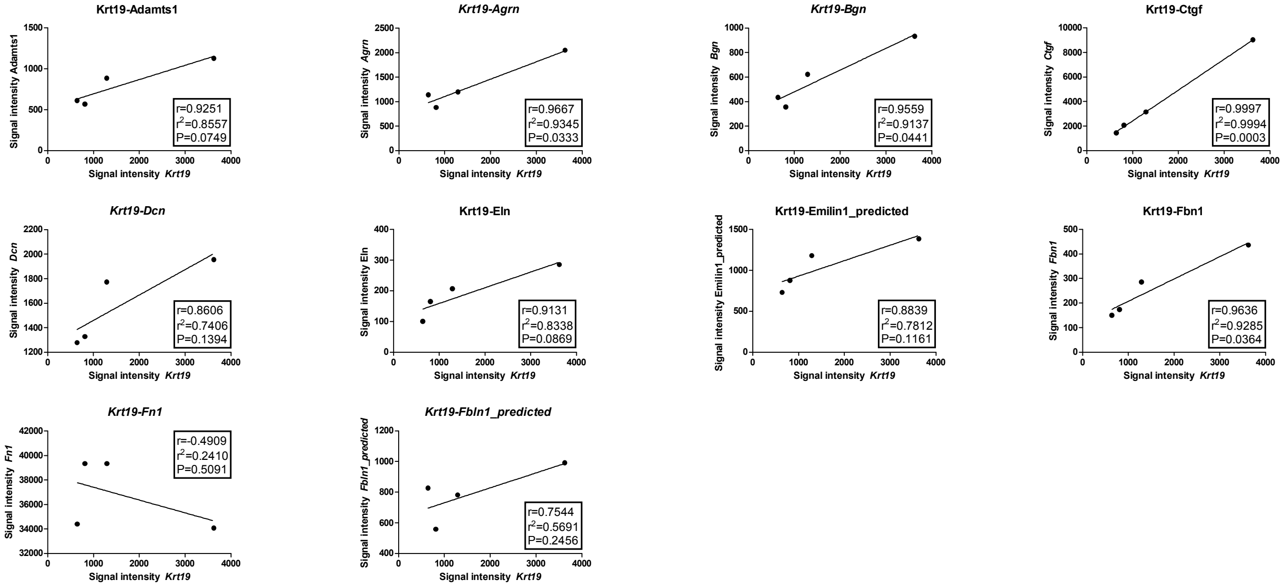

Des versus auxiliary proteins

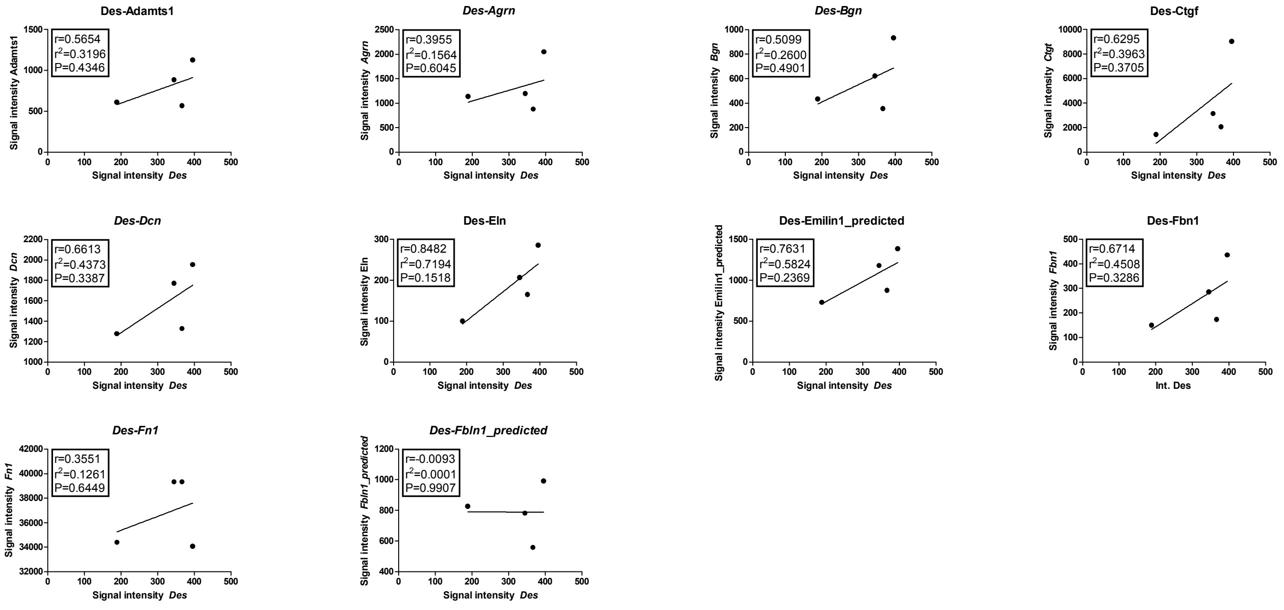

Additional File 2 Panel d
Auxiliary proteins ± SEM

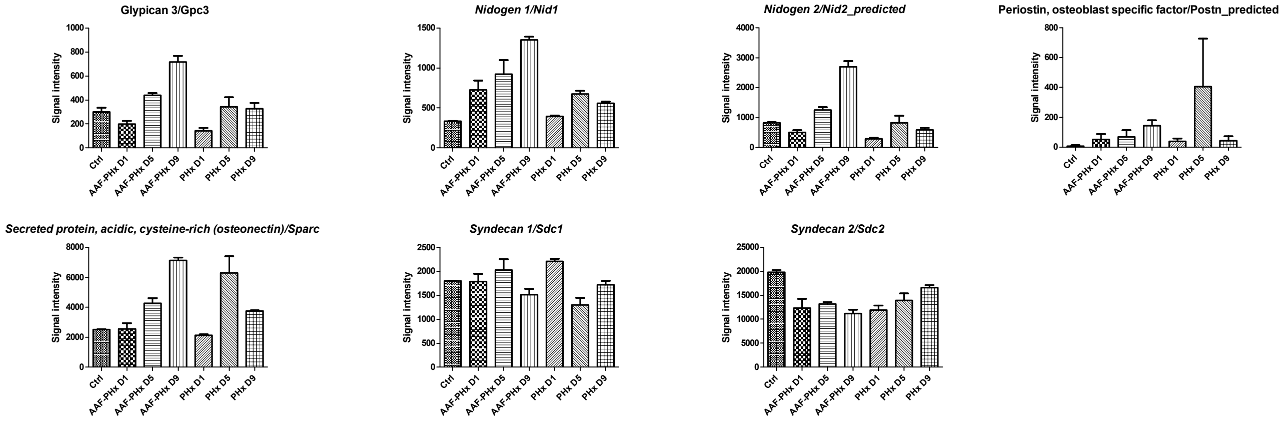

Krt19 versus auxiliary proteins

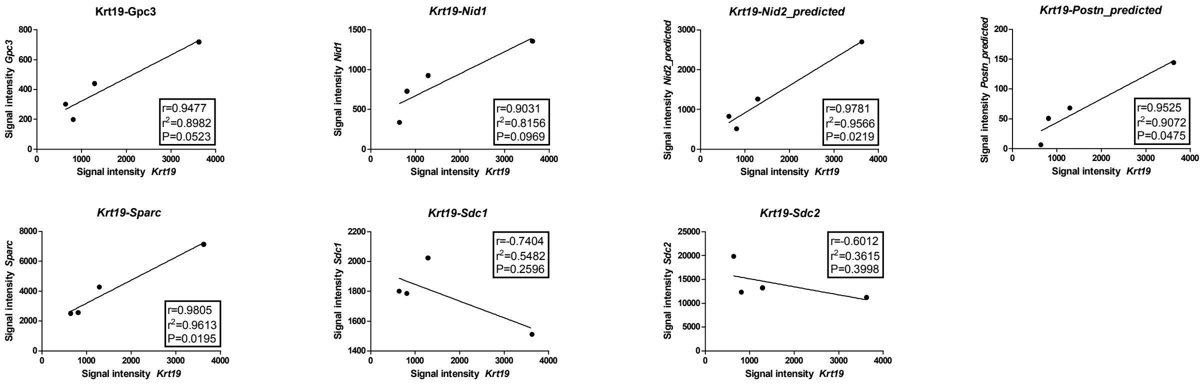

Des versus auxiliary proteins

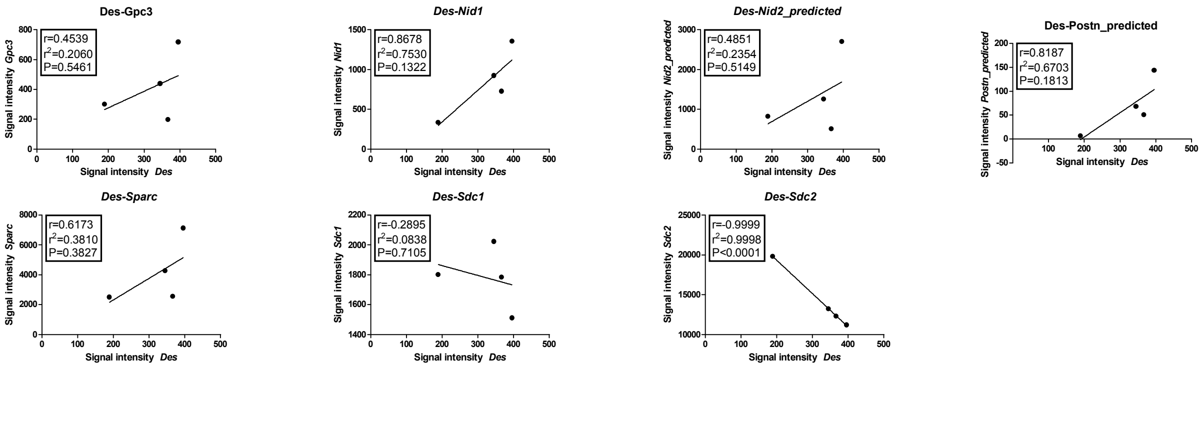

Additional File 2 Panel e

Collagens ± SEM

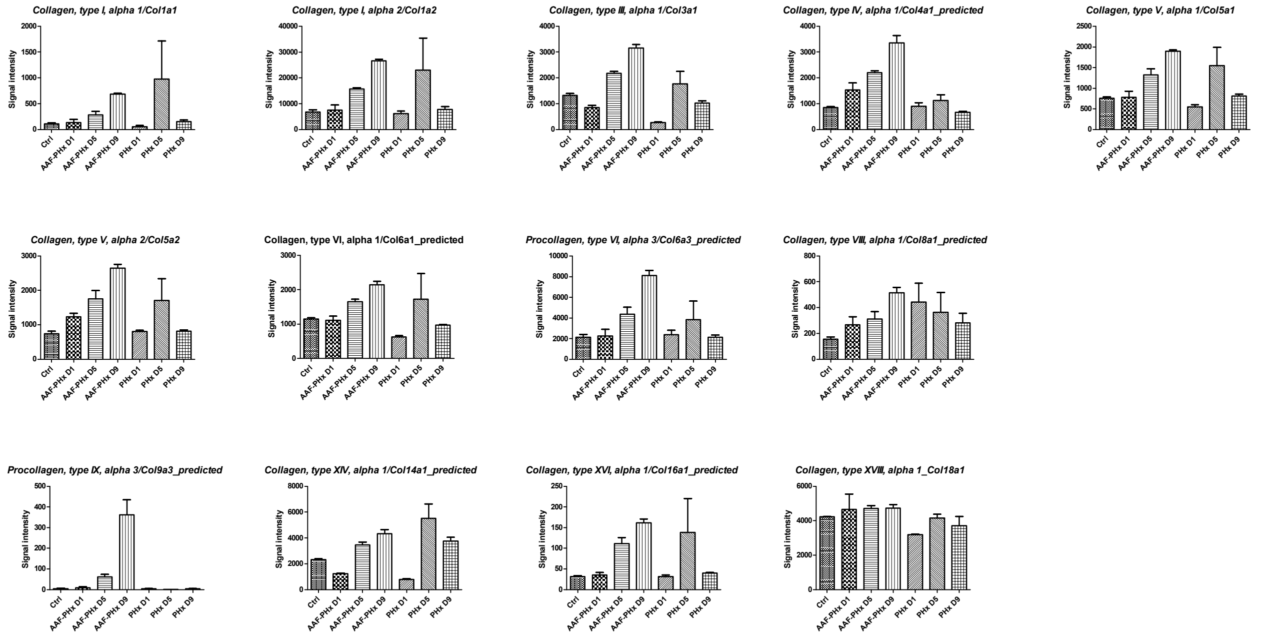

Krt19 versus collagens

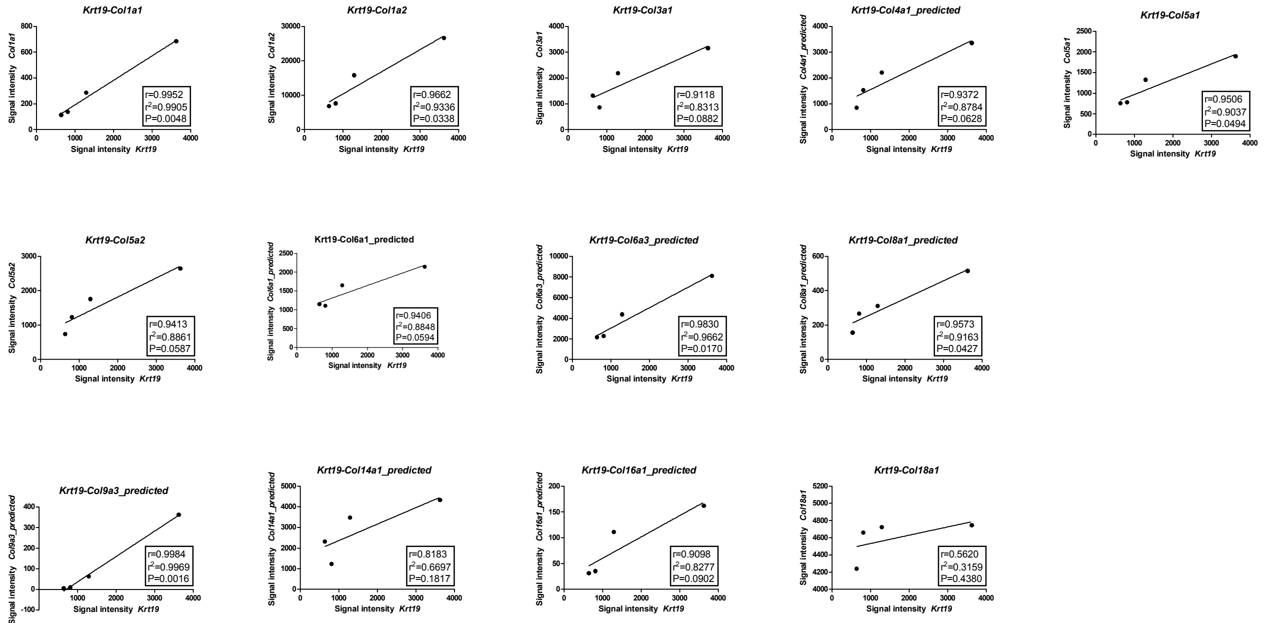

Des versus collagens

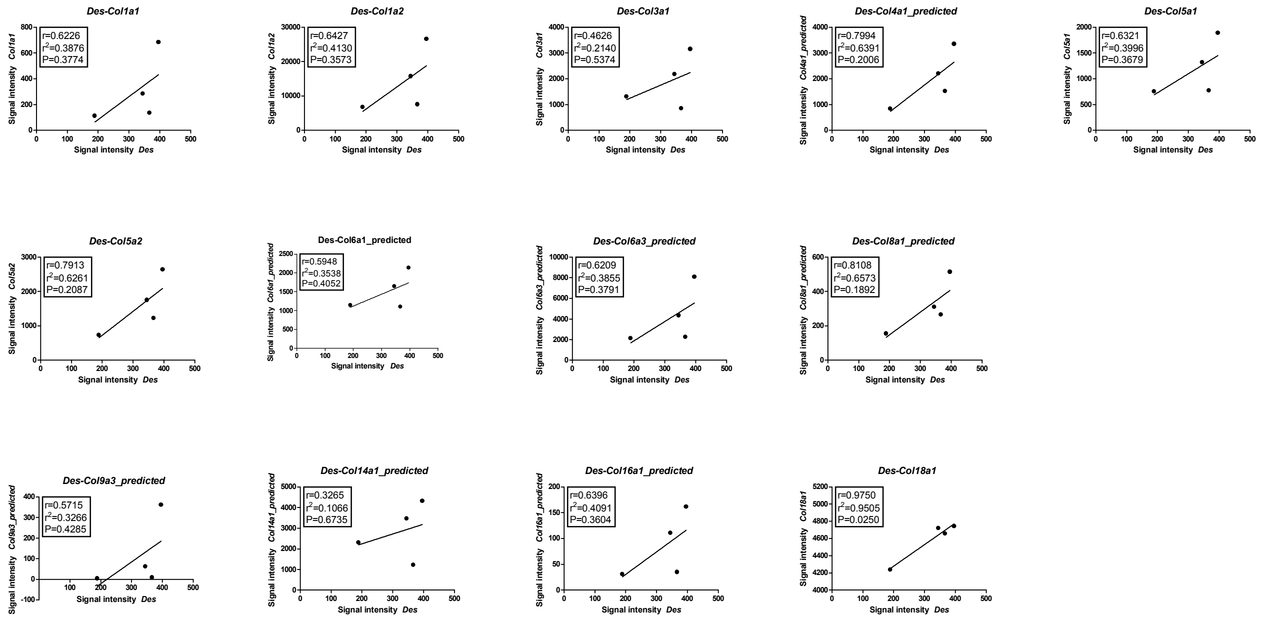

Additional File 2 Panel f

Laminins ± SEM

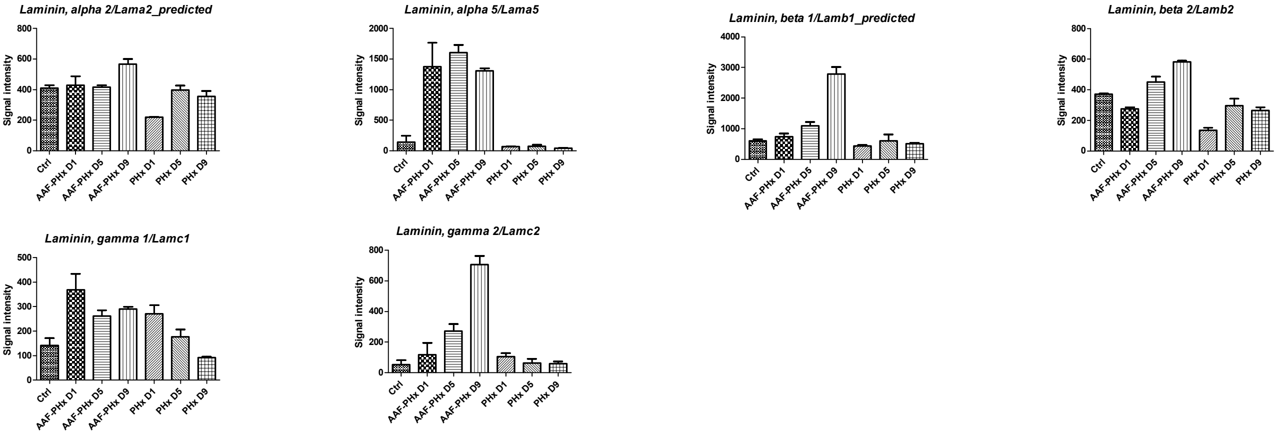

Krt19 versus laminins

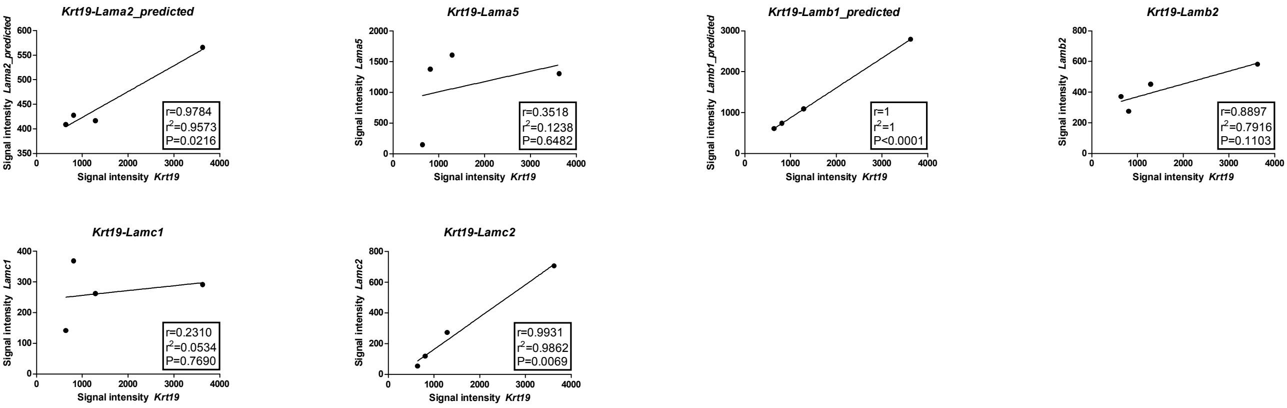

Des versus laminins

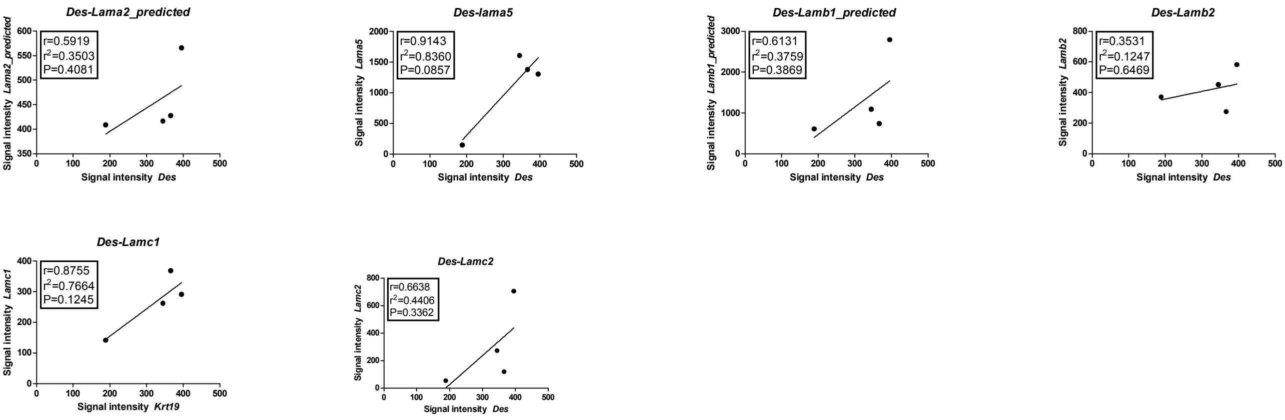

Additional File 2 Panel g

Integrins ± SEM

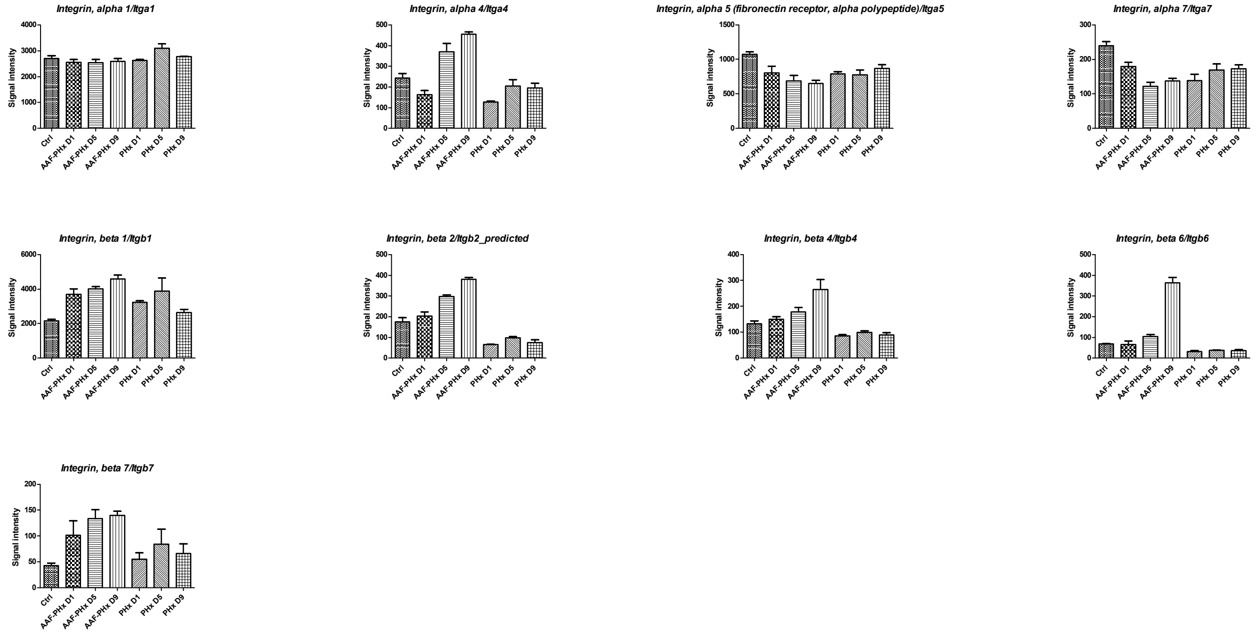

Krt19 versus integrins

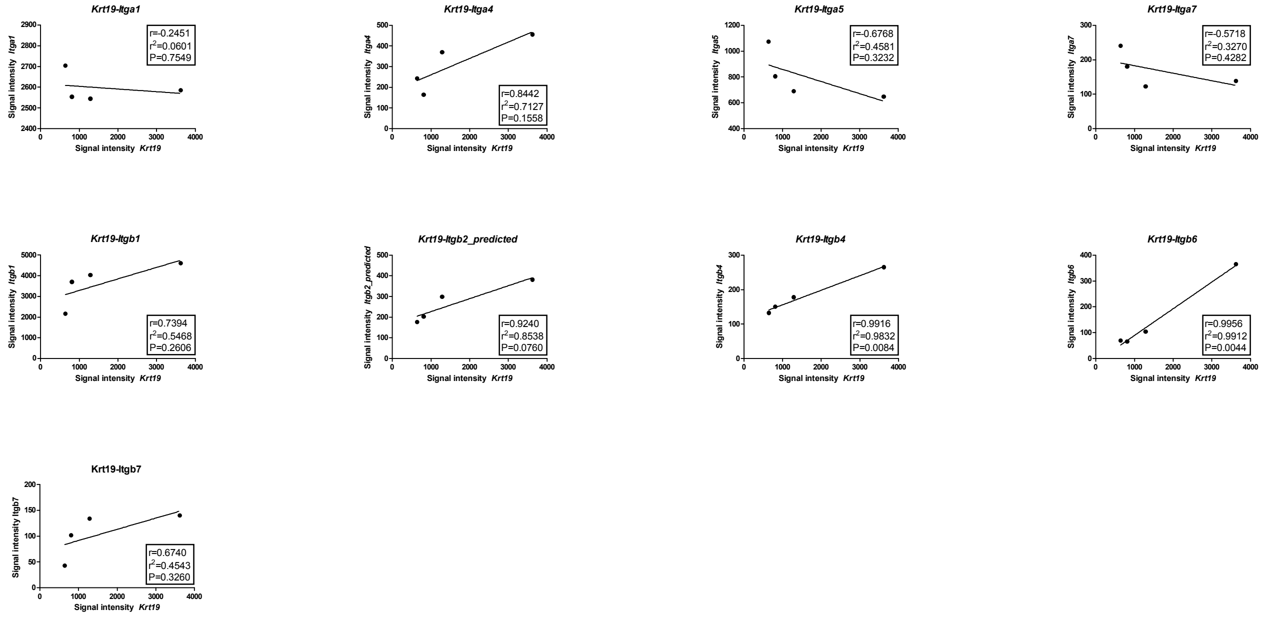

Des versus integrins

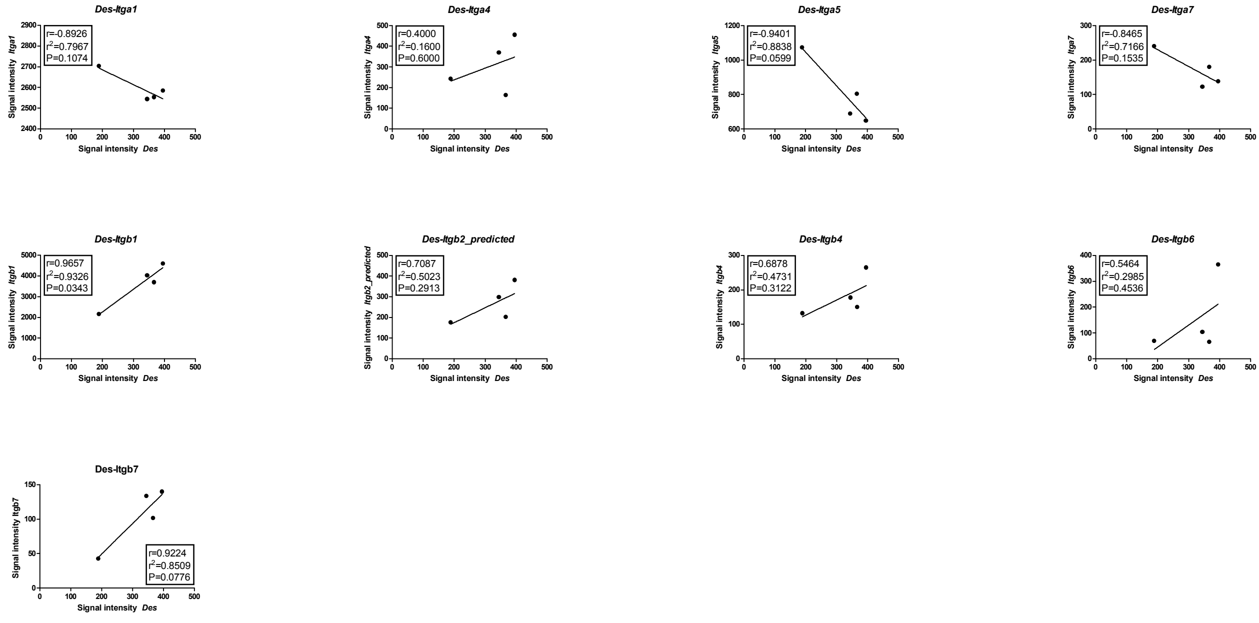

Additional File 2 Panel h

Matrix metallo peptidases and inhibitors ± SEM

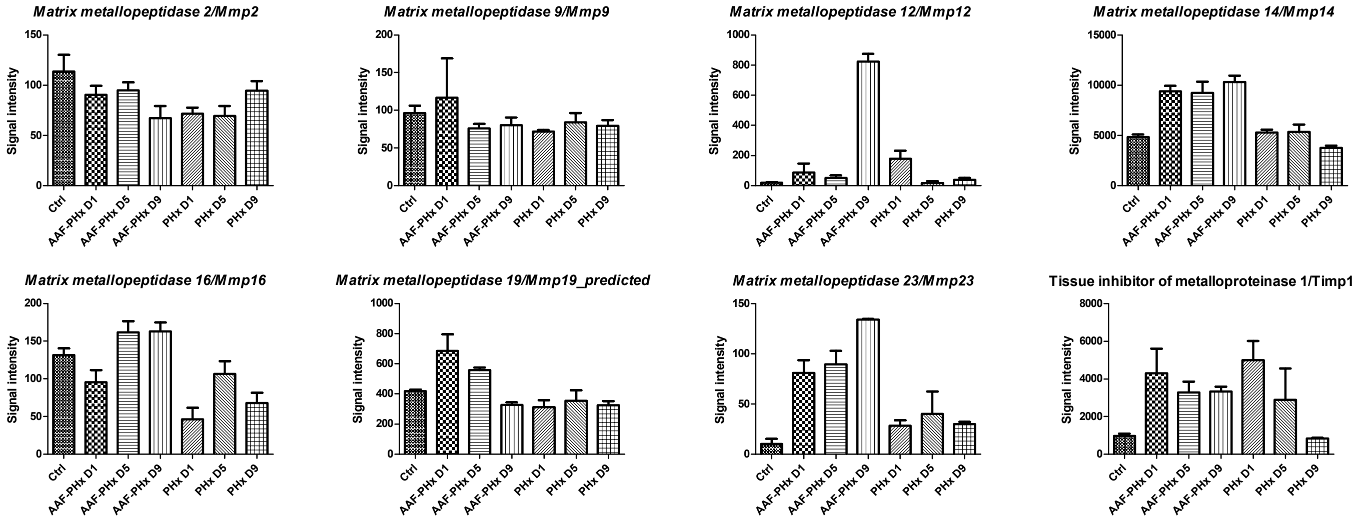

Krt19 versus Matrix metallo peptidases and inhibitors

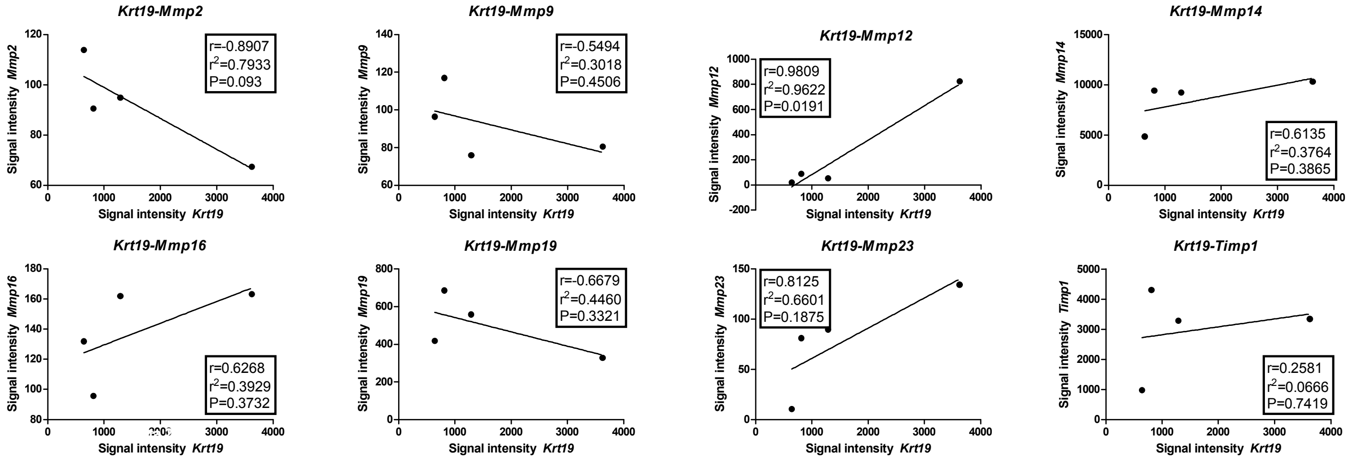

Des versus Matrix metallo peptidases and inhibitors

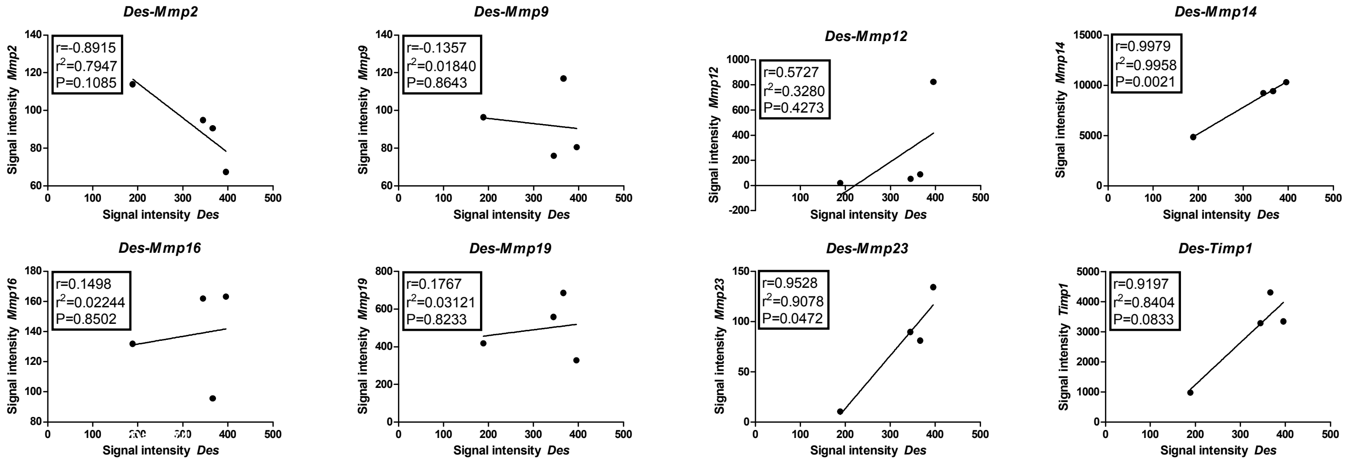

Additional File 2 Panel i

Matriptase network components ± SEM

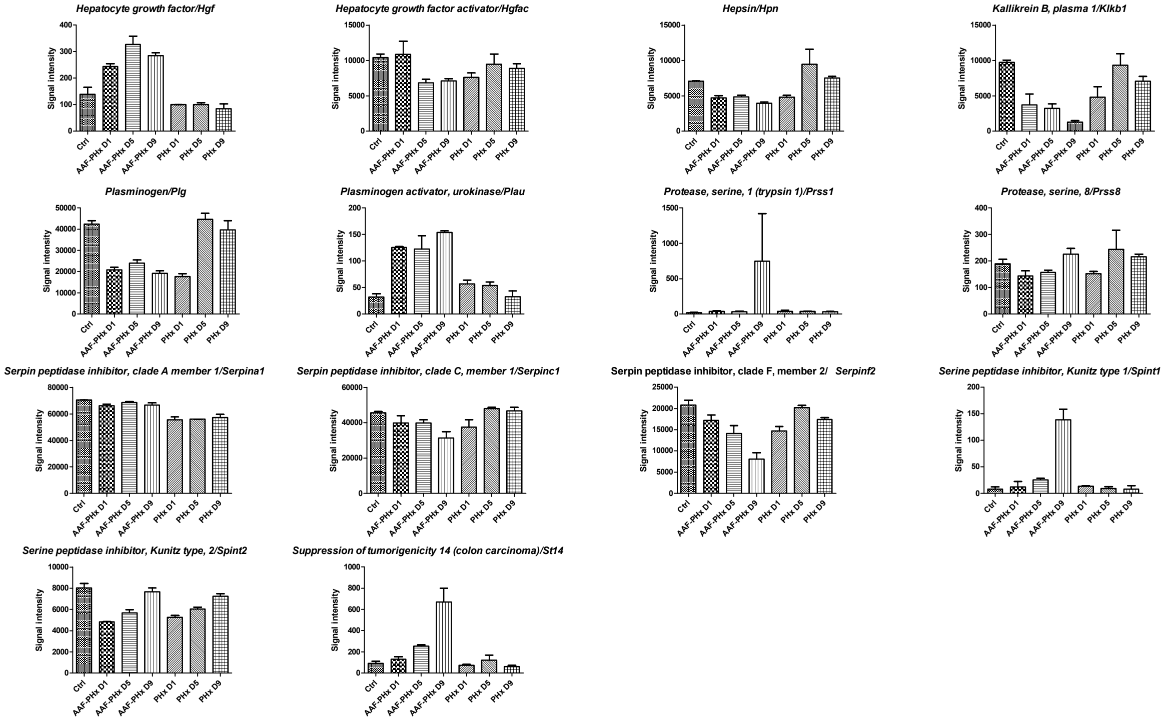

Krt19 versus matriptase network components

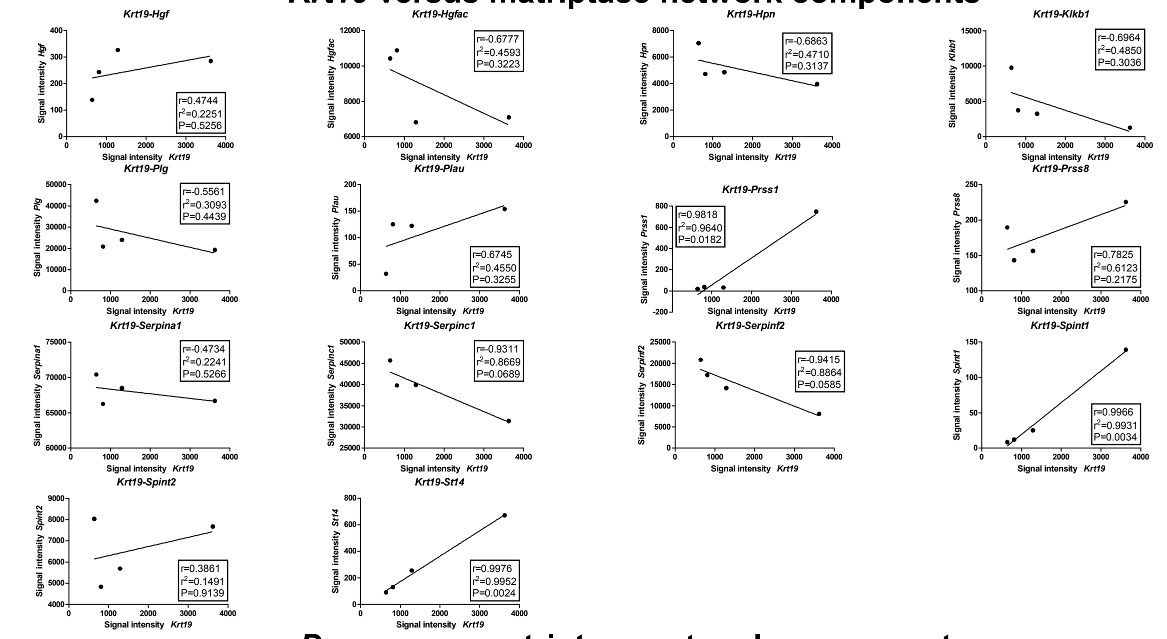

Des versus matriptase network components

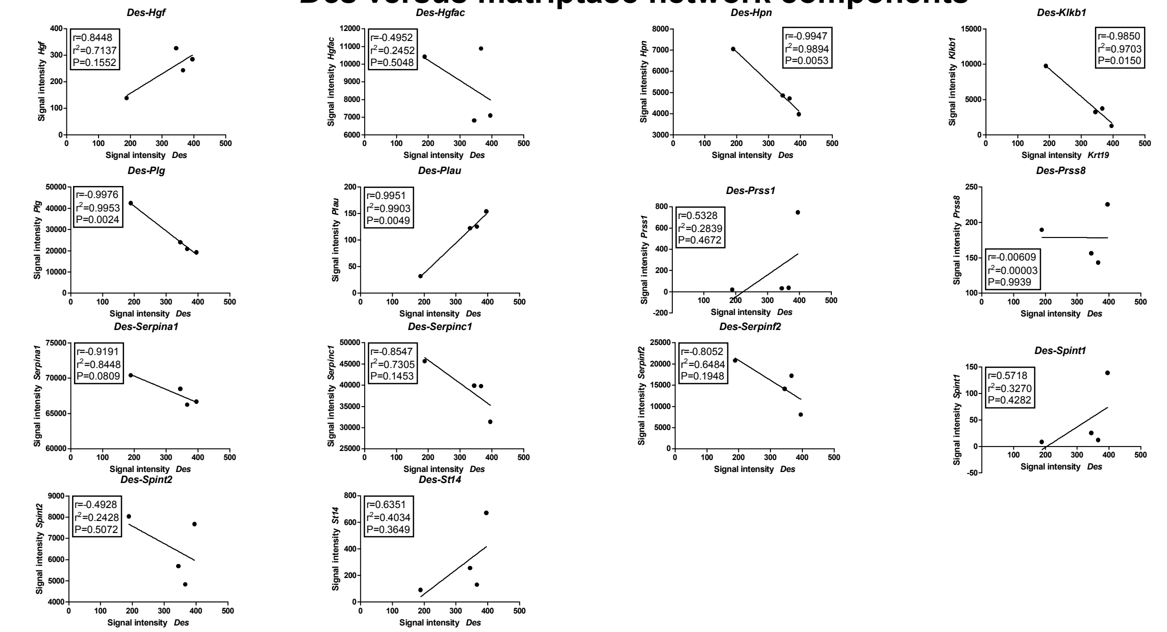

Supplement: Additional file 2 — Graphic illustration of signal intensities for genes encoding ECM constituents differentially expressed among classes in a first tier or second tier of defense using the PHx and AAF/PHx protocols as model systems. Presentations of selected gene expression data from livers of control rats and rats subjected to the 2-acetylaminofluorene/70% partial hepatectomy protocol of progenitor cell activation. Transcripts are categorized according to function: (a) epithelial (oval) cell markers; (b) mesenchymal cell markers; (c,d) auxiliary proteins; (e) collagens; (f) laminins; (g) integrins; (h) matrix metallo peptidases and inhibitors; (i) and matriptase network components. Linear regressions and correlation values were calculated between signal intensities for transcripts versus the hepatic progenitor cell marker Krt19 (Krt19) or the mesenchymal cell marker desmin (Des). Transcripts with signal intensities below 75 were disregarded. Data are presented as ‘signal intensity’ ± standard error of the mean). [file 1755-1536-6-21-S2.pdf]
