# Supplementary figures and images for: Molecular constituents of the extracellular matrix in rat liver mounting a hepatic progenitor cell response for tissue repair
Source: Fibrogenesis Tissue Repair. 2013 Dec 20;6:21. doi: 10.1186/1755-1536-6-21 (PMC3892118; doi:10.1186/1755-1536-6-21)

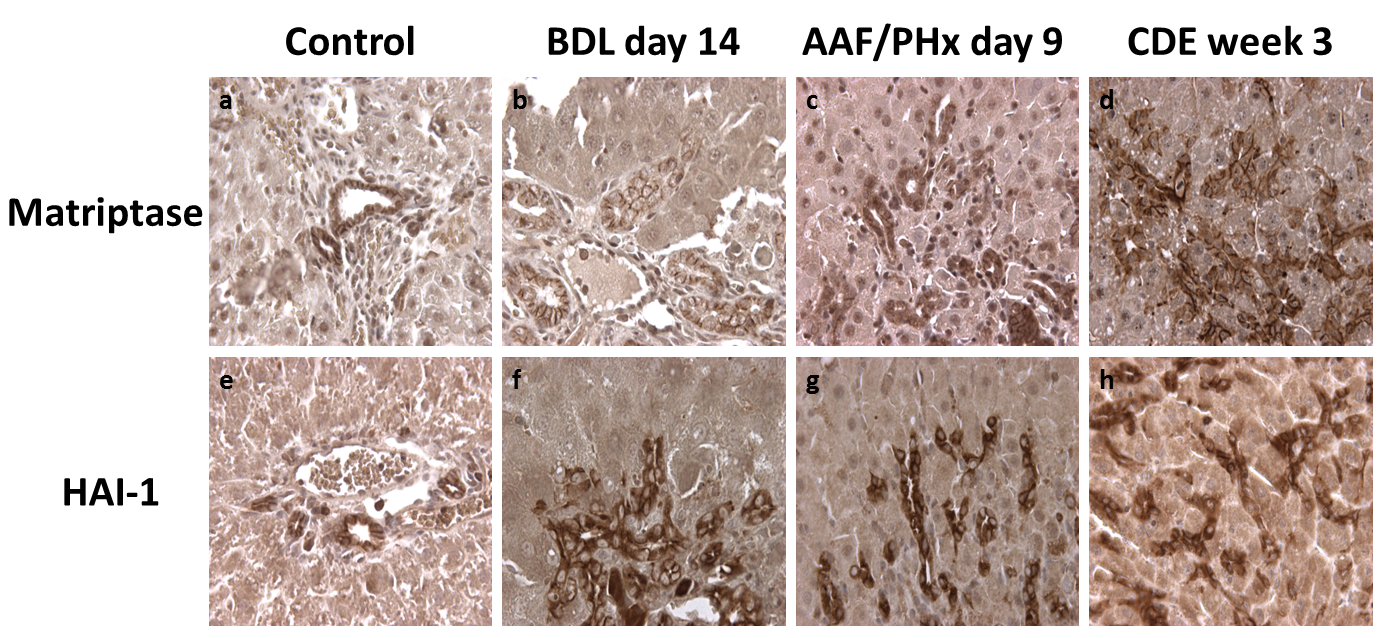

Supplement: Additional file 3 — Matriptase and HAI-1 highlights the cholangiocytic or biliary lineage. Photomicrographs showing expression of (a) matriptase and (e) its cognate inhibitor HAI-1 in the epithelial cells of the biliary tree in control liver. Correspondingly, (b-d) matriptase and (f-h) HAI-1 mark the epithelial cells in the biliary tree of (b,f) bile duct ligated rat liver and the HPC response in two protocols of hepatic progenitor cell activation; (c,g) the 2-acetylaminofluorene/70% partial hepatectomy and (d,h) choline-supplemented ethionine-deficient diet, respectively. Magnification 40×. [file 1755-1536-6-21-S3.tiff]

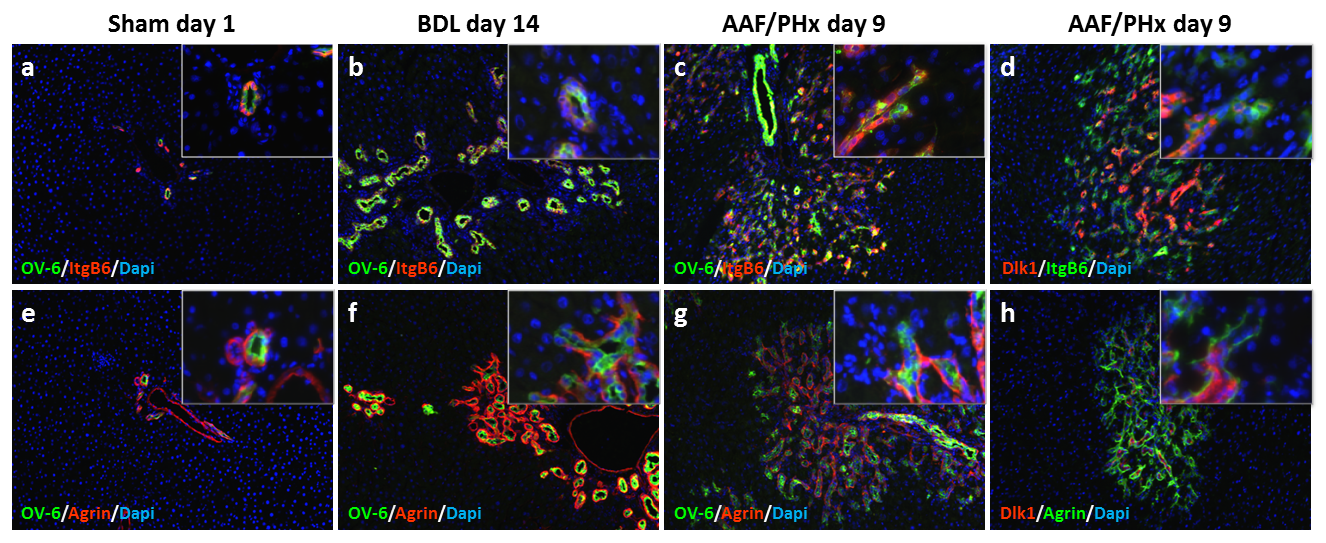

Supplement: Additional file 6 — Expression of investigated proteins is similar across rat hepatic injury protocols. (a-d) Integrin-β6 and (a,b) OV6/Krt19 mark epithelial cells in the biliary tree in (a) sham operated and (b) bile duct ligated (BDL) rats, respectively. (c,d) Similar expression for integrin-β6 is observed in the 2-acetylaminofluorene/70% partial hepatectomy (AAF/PHx) protocol with a hepatic progenitor cell response. (e) Agrin highlights the portal vein endothelia, portal artery and encloses the biliary tree in sham operated liver. In both (f) the BDL and (g) AAF/PHx- protocols, agrin escorts OV-6/Krt19-positive cells. (inserts in f,g). OV6/Krt19- or Integrin-β6-positive terminal cells in the canal of Hering penetrating into the lobules frequently extend beyond deposited agrin. (h) Dlk1-positive subpopulations in HPC response are enclosed by agrin, (insert in h) the latter forming tubular structures depicting the extent of the ductular reactions. Magnification 10×, inserts 40×. [file 1755-1536-6-21-S6.tiff]
